# Activation of the Pleiotropic Drug Resistance Pathway Can Promote Mitochondrial DNA Retention by Fusion-Defective Mitochondria in *Saccharomyces cerevisiae*

**DOI:** 10.1534/g3.114.010330

**Published:** 2014-05-06

**Authors:** Nebibe Mutlu, Görkem Garipler, Emel Akdoğan, Cory D. Dunn

**Affiliations:** Department of Molecular Biology and Genetic, Koç University, Sarıyer, İstanbul, 34450, Turkey

**Keywords:** petite-negative, mitochondrial genome, bulk segregant analysis, mitochondrial shape, drug resistance

## Abstract

Genetic and microscopic approaches using *Saccharomyces cerevisiae* have identified many proteins that play a role in mitochondrial dynamics, but it is possible that other proteins and pathways that play a role in mitochondrial division and fusion remain to be discovered. Mutants lacking mitochondrial fusion are characterized by rapid loss of mitochondrial DNA. We took advantage of a petite-negative mutant that is unable to survive mitochondrial DNA loss to select for mutations that allow cells with fusion-deficient mitochondria to maintain the mitochondrial genome on fermentable medium. Next-generation sequencing revealed that all identified suppressor mutations not associated with known mitochondrial division components were localized to *PDR1* or *PDR3*, which encode transcription factors promoting drug resistance. Further studies revealed that at least one, if not all, of these suppressor mutations dominantly increases resistance to known substrates of the pleiotropic drug resistance pathway. Interestingly, hyperactivation of this pathway did not significantly affect mitochondrial shape, suggesting that mitochondrial division was not greatly affected. Our results reveal an intriguing genetic connection between pleiotropic drug resistance and mitochondrial dynamics.

The abundance, shape, and size of mitochondria are determined by the rate of mitochondrial division and fusion. Both of these processes take place with the aid of GTPases that are conserved across the eukaryotic domain. The dynamin family member Dnm1p is key for mitochondrial division in *Saccharomyces cerevisiae* (Lackner and Nunnari 2009; Kageyama *et al.* 2011; Chan 2012; Westermann 2013). Dnm1p forms rings and spirals that are recruited to the sites of mitochondrial division by specific adaptor proteins (Ingerman *et al.* 2005; Naylor *et al.* 2006). These adaptor proteins differ among organisms but in *S. cerevisiae* consist of the soluble WD40-domain containing proteins Mdv1p and Caf4p and the membrane receptor Fis1p (Fekkes *et al.* 2000; Mozdy *et al.* 2000; Tieu and Nunnari 2000; Tieu *et al.* 2002; Cerveny and Jensen 2003; Griffin *et al.* 2005). Much evidence currently supports a model in which cooperative GTP hydrolysis by Dnm1p oligomers at the mitochondrial surface squeezes the mitochondrial tubule, thereby forcing mitochondrial scission (Mears *et al.* 2011).

Recent studies have illuminated numerous ways in which the process of mitochondrial division can be controlled by the cell. For example, phosphorylation of the human Dnm1p ortholog Drp1 by protein kinase A inhibits mitochondrial division and protects neurons under stress conditions (Merrill *et al.* 2011). In addition, Drp1 can be SUMOylated (Braschi *et al.* 2009; Zunino *et al.* 2009), S-nitrosylated (Cho *et al.* 2009), or ubiquitylated (Karbowski *et al.* 2007), all of which may bear upon the mitochondrial fission process. Moreover, association with another organelle system, the endoplasmic reticulum (ER), can determine the location of mitochondrial scission (Friedman *et al.* 2011). These exciting findings raise the possibility that additional genes and pathways that influence the rate of mitochondrial division might be identified and mechanistically studied using *S. cerevisiae*.

Like mitochondrial division, mitochondrial fusion is dependent upon dynamin-family GTPases. These fusion-mediating proteins reside at both the outer membrane (OM) and the inner membrane (IM) of mitochondria (Hoppins and Nunnari 2009; Chan 2012). In *S. cerevisiae*, Fzo1p allows OM tethering and fusion (Meeusen *et al.* 2004), and Mgm1p is thought to catalyze IM fusion (Meeusen *et al.* 2006). Ugo1p connects the OM and IM fusion machineries (Wong *et al.* 2003; Sesaki and Jensen 2004). Mutations blocking mitochondrial fusion result in fragmentation of mitochondrial tubules and, for reasons that are not yet understood, mitochondrial DNA (mtDNA) loss (Hermann *et al.* 1998; Rapaport *et al.* 1998). However, this mitochondrial fragmentation is dependent upon the mitochondrial division machinery; cells lacking both the ability to fuse mitochondria and the capacity to divide mitochondria are able to maintain both tubular mitochondrial morphology and mtDNA (Bleazard *et al.* 1999; Sesaki and Jensen 1999). Selection for mutants that maintain mtDNA when mitochondrial fusion is blocked has successfully revealed mitochondrial division machinery components (Fekkes *et al.* 2000; Mozdy *et al.* 2000; Tieu and Nunnari 2000). However, selection for mtDNA retention was previously performed using nonfermentable medium, potentially excluding suppressors that block oxidative phosphorylation or otherwise inhibit proliferation under those specific culture conditions.

In this study, we applied a novel selection approach to search for new mutations that would allow cells unable to fuse mitochondria to maintain mtDNA. By sequencing the entire genomes of suppressor-containing isolates, we found that dominant mutations activating the pleiotropic drug resistance (PDR) pathway can allow cells lacking mitochondrial fusion components to keep the mitochondrial genome, providing additional evidence of a functional relationship between the PDR pathway and mitochondrial biogenesis.

## Materials and Methods

### Yeast strains and culture conditions

Yeast media were prepared as described in Adams *et al.* (1997). Gene disruptions were performed as detailed in Sikorski and Hieter (1989) and Taxis and Knop (2006). Strains were cultured at 30° except where otherwise indicated. The genotypes of strains used in the course of this study, along with construction details, are provided in Supporting Information, Table S1. Oligonucleotides used during this study are listed in Table S2. Ethidium bromide (Thermo-Fisher Scientific, Waltham, MA) was used at a concentration of 25 µg/mL to destroy mtDNA. Cycloheximide (CHX; Sigma-Aldrich, St. Louis, MO) was used at a concentration of 10 µg/mL for plasmid counterselection on yeast extract peptone dextrose broth (YEPD) medium, 3 µg/mL for plasmid counterselection on yeast extract peptone 3% glycerol+3% ethanol (YEPGE) medium, and at 0.2 µg/mL in YEPD medium to test for activation of the PDR pathway. Ketoconazole (Tokyo Chemical Industry Co., Tokyo, Japan), another PDR substrate, was used at a concentration of 2 µg/mL in YEPD. To disrupt the actin cytoskeleton in logarithmic-phase cultures, latrunculin A (Santa Cruz Biotechnology, Dallas, TX) was used at a concentration of 10 µM in SD medium lacking leucine (SD-Leu), following the procedure of Hammermeister *et al.* (2010). Serial dilution assays were performed as in Garipler *et al.* (2014).

### Plasmid construction and acquisition

To generate p*FZO1-CYH2-TRP1* plasmid b19, the *FZO1* open reading frame, along with 476 bp of upstream and 508 bp of downstream sequence, was amplified by polymerase chain reaction (PCR) with primers 1 and 2, digested with *Not*I, and ligated into *Not*I-linearized pKS1 (Ryan *et al.* 1998). For construction of p*MGM1-CYH2-TRP1* plasmid b86, the *MGM1* coding region, together with 500 bp of upstream sequence and 108 bp of downstream sequence, was amplified by PCR with primers 42 and 43, digested with *Xho*I and *Not*I, and ligated into *Xho*I/*Not*I-cut pKS1. To construct plasmids containing *PDR1* alleles, the *PDR1* open reading frame, along with 556 bp of upstream and 284 bp of downstream sequence, was amplified by PCR with primers 459 and 460. This PCR product was digested using *Xba*I and *Not*I then ligated into pRS313 (Sikorski and Hieter 1989) subjected to cleavage with the same enzymes. Plasmids b60, b61, b62, b63, b64, and b65 contained *PDR1* alleles amplified from strains CDD71, CDD91, CDD95, CDD98, CDD99, and CDD100, respectively. pHS12, a *LEU2*-containing plasmid expressing Cox4(1-21)-green fluorescent protein (GFP) (Sesaki and Jensen 1999) was a kind gift of Dr. Hiromi Sesaki, Johns Hopkins School of Medicine. pM390, a ARS/CEN-containing plasmid incorporating the *LEU2* gene and expressing an Abf2-GFP fusion protein, was generously provided by Prof. Robert Jensen, Johns Hopkins School of Medicine.

### Selection for suppressors of *fzo1∆ aac2∆* synthetic lethality (*sfa* alleles)

Individual colonies of strain CDD71 were patched to YEPD medium and either left untreated (potential *sfa* isolates 1−22) or irradiated for 2 s at 312 nm with a Herolab UVT-20M, which provided a lethality rate of 74% (potential *sfa* isolates 23−265). We note that *sfa* is denoted in lowercase, because the dominance of characterized mutations was not established until after mutation identification. Patches were replica-plated to YEPD medium containing 10 µg/mL CHX then replica plated again to the same medium. A single cycloheximide-resistant (CHX^R^) clone was isolated from each individual patch. To test for a CHX^R^ phenotype caused by mutation of the plasmid borne *CYH2* gene, isolates were replica-plated to synthetic dextrose medium lacking tryptophan (SD-Trp). Those isolates proliferating on SD-Trp medium were assumed to contain a mutated *CYH2* allele within the b19 plasmid and were discarded.

The remaining isolates were replica plated to *fzo1∆ aac2∆ cyh2* cells of the opposite mating type that were deleted of *DNM1* (CDD72), *MDV1* (CDD73), or *FIS1* (CDD74) for the purpose of complementation testing. We note that *fzo1∆ aac2∆* cells exhibit a “leaky” phenotype: some *fzo1∆ aac2∆* mutant clones keep enough mtDNA to allow very slow proliferation on fermentable medium. Noncomplementation, during our triage of *sfa* alleles, was defined as the strong suppression of mtDNA loss from *fzo1∆ aac2∆* cells at a level typical of mutations affecting known mitochondrial division components. Results from these crosses suggested that we had recovered 64 *dnm1* alleles, 14 *mdv1* alleles, and 11 *fis1* alleles. Twenty-nine *sfa* allele-containing isolates either provided inconsistent results among different complementation tests or, alternatively, demonstrated noncomplementation of the suppressor mutation by multiple deletions of division components. The isolation of mitochondrial division-blocking alleles that exhibit unlinked noncomplementation in combination with other division-hampering alleles has been described previously (Tieu and Nunnari 2000). Six other isolates failed to mate but were discarded after detecting the presence of *FZO1* by PCR. Two other mutations were clearly and strongly dominant and were not studied further. We initially overlooked the at least partial dominance of the mutations that were later characterized in this study, because the strength of suppression in diploid cells generated during complementation testing appeared weak compared with the suppression conferred by homozygous deletion of known division components.

Twelve remaining *fzo1∆ aac2∆ cyh2 sfa* isolates were mated to *cyh2* strain CDD51 transformed with plasmid b19 and lacking functional mtDNA following two days of culture in the presence of 25 µg/mL ethidium bromide (Goldring *et al.* 1970). All resulting diploids proliferated on YEPGE medium, suggesting that *sfa* mutations had allowed maintenance of mtDNA in the absence of *FZO1* and, therefore, *sfa* alleles do not suppress the petite-negative phenotype of *aac2∆* cells. For many *sfa* alleles, including the *PDR1-249* allele most prominently described in this work, absence of *FZO1* coding sequence was also confirmed in the initial *fzo1∆ aac2∆ sfa* isolate by PCR before further characterization.

Next, *fzo1∆/FZO1aac2∆ /AAC2cyh2/cyh2 sfa/SFA* p*FZO1-CYH2-TRP1* strains generated by the aforementioned mating were sporulated, and haploid *fzo1∆ aac2∆ cyh2 sfa* progeny were tested for survival after loss of plasmid b19 on YEPD medium containing CHX. During our analysis comparing proliferation rates of haploid progeny after loss of *FZO1*, the suppressive phenotype of four presumptive *sfa* alleles did not appear to be mediated by a single allele segregating in a Mendelian fashion. Those four alleles were not studied further. However, later identification of one allele of *PDR3* as a *fzo1∆ aac2∆* suppressor raises the possibility that close linkage of *PDR3* to *AAC2* (Subik *et al.* 1986) may have confounded our analysis and that at least some of these four discarded *sfa* alleles could contain mutations at the *PDR3* locus. The other eight alleles exhibited a segregation pattern characteristic of a single mutation existing on a nuclear chromosome.

### Bulk segregant analysis and next-generation sequencing

To identify seven of the *sfa* mutations allowing mtDNA retention by cells lacking *FZO1*, bulk segregant analysis (Michelmore *et al.* 1991) was followed by next-generation sequencing. From *fzo1∆/FZO1aac2∆/AAC2cyh2/cyh2 sfa/SFA* diploids derived from strains CDD91, CDD95, CDD98, CDD99, CDD100, CDD104, and CDD105, suppressor-containing and suppressor-lacking meiotic products were combined into pools. At least three separate isolates were combined in each pool. Similarly, seven pools consisting of isolates lacking suppressors and derived from the same seven diploids were analyzed. For each genomic DNA preparation, equal volumes of saturated cultures of each *fzo1∆ aac2∆* isolate either lacking or containing a *sfa* allele were mixed, and genomic DNA was extracted essentially as in Looke *et al.* (2011).

Paired-end library preparation was performed at the European Molecular Biology Laboratory (EMBL) Genomics Core Facility (Heidelberg, Germany) using the Illumina protocol for preparation of genomic DNA sequencing libraries. Library fragments averaging 300 bp (standard deviation of 25 bp) were sequenced on the Illumina HiSeq platform to a read length of 100 bp. Sequencing reads have been deposited into the Sequence Read Archive of the National Center for Biotechnology Information under accession number PRJNA229450.

### Bioinformatic analysis of genomic sequence

Read mapping against the sacCer3 reference genome for *S. cerevisiae* was performed at EMBL and data resulting from the use of the Illumina ELAND algorithm were converted to BAM format. The sacCer3 reference genome was also used for all subsequent analysis. BAM files were sorted using samtools version 0.1.18 (Li *et al.* 2009). Next, the mpileup function of samtools was used to compare, for each suppressor mutation: the gDNA pool carrying the *sfa* allele, the gDNA pool not carrying the *sfa* allele, and, for subtraction of sequence differences between the S288C and W303 backgrounds, W303 control strain CDD101. VarScan version 2.3.5 (Koboldt *et al.* 2009) was used to analyze each mpileup output file to locate base pair changes that were private to gDNA pools containing *sfa* alleles. Minimum supporting reads necessary to identify a variant was set to 10, and minimum frequency for an allele to be considered homozygous was set to 0.90. VarScan output used to identify mutant bases associated with each characterized *sfa* allele is provided as File S1.

### Fluorescence microscopy

Microscopy was accomplished using the equipment described in (Garipler and Dunn 2013). MitoTracker Green FM staining (Molecular Probes, Eugene, OR) was performed in culture medium for 20 min at a concentration of 500 nM before examination of mitochondrial morphology.

### Southern blotting

Total genomic DNA was isolated essentially as in Looke *et al.* (2011). Ten micrograms of genomic DNA were digested by *Eco*RV, electrophoresed through a 0.8% agarose gel, then transferred to a nylon membrane and covalently bound to the membrane using a CL-508G UV cross-linker (UVItec Ltd., Cambridge, UK). A probe for quantification of nuclear DNA was synthesized by PCR using primers 537 and 538 and a genomic DNA template, thereby amplifying a 501 bp portion of the *TDH1* open reading frame predicted to hybridize to a 3364 bp fragment of *Eco*RV-cut nuclear DNA. A probe for quantification of mtDNA was generated by PCR using primers 535 and 536 and a genomic DNA template to amplify a 563 bp portion of the *COX3* open reading frame that was predicted to hybridize to a 8425 bp fragment of *Eco*RV-cut mtDNA. PCR probes were labeled with ^32^P using the Prime-a-Gene Labeling System (Promega, Madison, WI) and radiolabeled products were hybridized to target sequences in PerfectHyb Plus buffer at 68° using manufacturer’s instructions (Sigma-Aldrich, St. Louis, MO). A high stringency wash (0.5X saline-sodium citrate (SSC) buffer, 0.1% sodium dodecyl sulfate) was performed after hybridization with *COX3* probe. Following phosphorimaging using a Perkin Elmer Cyclone (Waltham, MA), the same membrane was probed for *TDH1* and a low stringency wash (2X SSC, 0.1% SDS) was carried out before signal detection.

## Results

### A selection for mutations preventing mtDNA loss from cells lacking mitochondrial fusion machinery

Selection of mutations affecting mitochondrial division has previously depended upon the recovery of respiring cells from nonfermentable medium (Fekkes *et al.* 2000; Mozdy *et al.* 2000; Tieu and Nunnari 2000). With the goal of isolating new mitochondrial division machinery or proteins and pathways impinging upon the mitochondrial fission process, we similarly asked that fusion-incompetent cells maintain mtDNA, yet we performed our selection on glucose-containing medium. Our methodology took advantage of the petite-negative phenotype, or the inability of certain mutants to live in the absence of mtDNA, even upon fermentable medium (Chen and Clark-Walker 2000). Specifically, our selection scheme used a nuclear background lacking the major ATP/ADP antiporter of the mitochondrial IM, *AAC2*. Unlike wild-type *S. cerevisiae* cells, *aac2* mutants cannot proliferate in the absence of mtDNA (Kovacova *et al.* 1968), yet are inviable on nonfermentable medium (Kovac *et al.* 1967; Beck *et al.* 1968).

**Figure 1:**
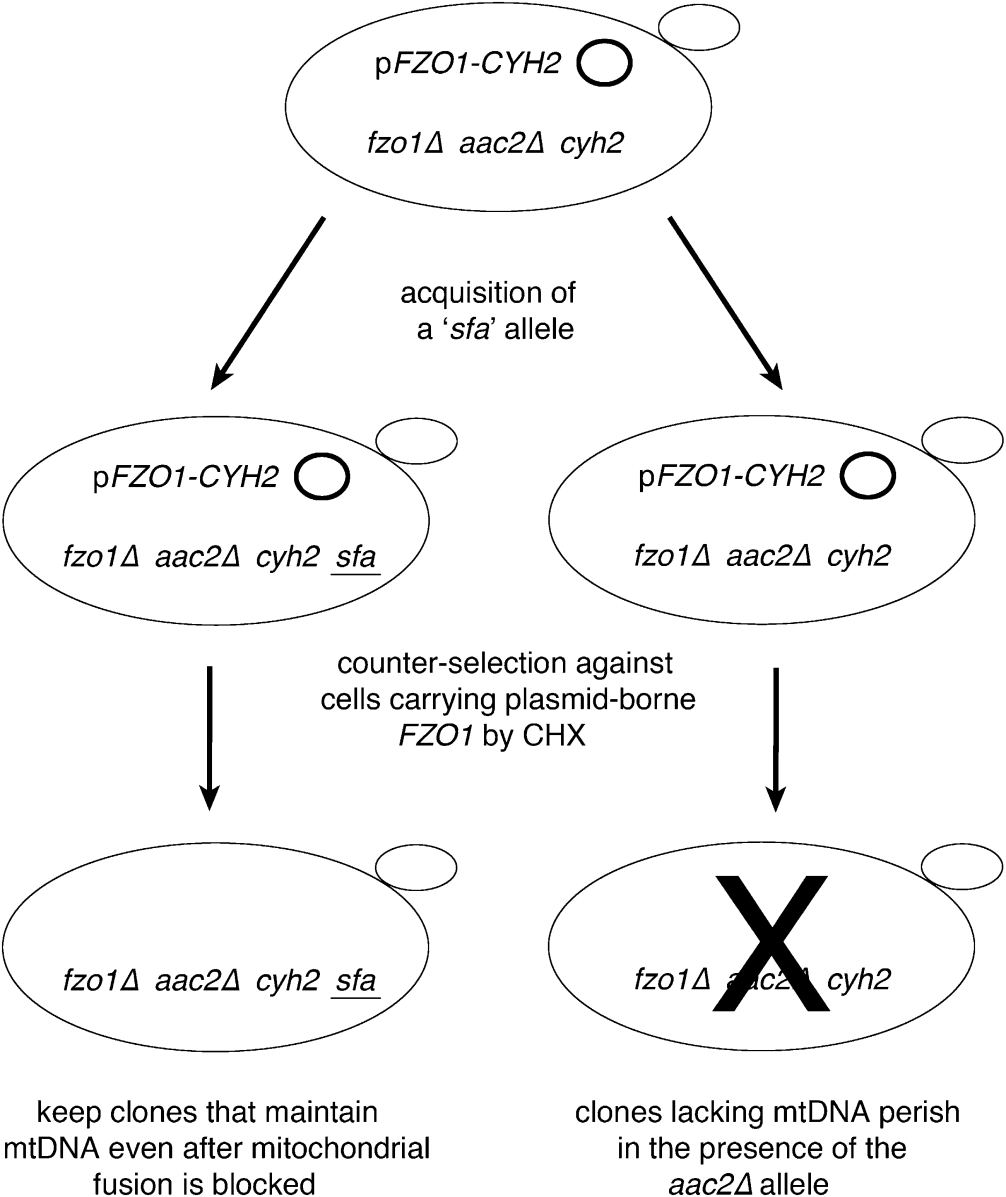
A scheme for identifying new genes playing a role in mitochondrial dynamics. Loss of the ATP/ADP antiporter Aac2p prevents survival after mitochondrial DNA (mtDNA) deletion (Kovacova *et al.* 1968). Blocking mitochondrial fusion by *FZO1* deletion in the presence of continued mitochondrial fission causes mtDNA loss (Bleazard *et al.* 1999; Sesaki and Jensen 1999). A strain was constructed (CDD71) carrying chromosomal *fzo1∆* and *aac2∆* mutations, in addition to a plasmid encoding *FZO1*. The *FZO1*-carrying plasmid was made counterselectable by a linked *CYH2* allele existing in the context of a chromosomal *cyh2* mutation that provides recessive cycloheximide (CHX) resistance (Sikorski and Boeke 1991). Strain CDD71 was either allowed to spontaneously acquire an *sfa* mutation, thereby permitting cells lacking *FZO1* to maintain mtDNA and to survive in the presence of the *aac2∆* mutation, or treated with ultraviolet light to generate such a mutation. Next, cells were replica-plated to medium containing 10 µg/mL CHX to counterselect for the *FZO1*-containing plasmid. Surviving colonies were isolated and further characterized as described in the *Materials and Methods*.

In the background of an *aac2∆* mutation, we removed the chromosomal copy of the *FZO1* gene, required for OM fusion, yet provided a plasmid encoding both *FZO1* and *CYH2*, allowing counter-selection by a high concentration of CHX in the presence of a chromosomal *cyh2* mutation (Sikorski and Boeke 1991) (Figure 1). We then patched individual colonies from the resulting *fzo1∆ aac2∆ cyh2* p*FZO1-CYH2* strain (CDD71) to YEPD medium. Some patches were subjected to ultraviolet (UV) irradiation to promote the generation of new mutations. Patches were then replica-plated to medium containing 10 µg/mL CHX to select for the absence of the *FZO1*-containing plasmid and thereby ensure cessation of mitochondrial fusion. Although *fzo1∆ aac2∆* cells proliferate very slowly, a single, rapidly growing colony was isolated from each of more than 200 patches, making certain the independence of any suppressor mutations. Changes allowing mtDNA maintenance were called “*sfa*” alleles as an abbreviation for “*suppressor of fzo1∆ aac2∆ synthetic lethality*.”

After confirming loss of the *FZO1*-containing plasmid, we attempted complementation tests to determine whether the newly isolated *sfa* mutations might be found in the *DNM1*, *MDV1*, or *FIS1* genes, the only three genes whose mutation has been demonstrated to allow mtDNA maintenance in the absence of mitochondrial fusion. Complementation testing suggested that we had isolated 64 *dnm1* mutations, 14 *mdv1* mutations, and 11 *fis1* mutations, and those alleles were not further characterized. Some *sfa* alleles exhibited noncomplementation when combined with more than one division component, perhaps due to the reported unlinked noncomplementation of certain mutations affecting mitochondrial division (Tieu and Nunnari 2000) and also were not studied further. Other *sfa* alleles provided variable results during complementation testing, and these were similarly discarded. In addition, two clearly dominant *sfa* alleles were obtained, but were discarded with the expectation that they might be mutations in the *DNM1* gene, in which such dominant-negative mutations have previously been isolated (Otsuga *et al.* 1998; Sesaki and Jensen 1999). Later, it became apparent that some *sfa* alleles originally classified as recessive suppressors were actually dominant in nature, yet provided weaker suppression than mutations of known division components. The dominant nature of several *sfa* alleles is further described below.

Twelve remaining *fzo1∆ aac2∆ sfa* isolates were crossed to a strain lacking mtDNA and carrying plasmid-borne *FZO1*. Resulting diploids were respiration competent, as expected for cells maintaining mtDNA, thereby demonstrating that *sfa* mutations do not suppress the petite-negative phenotype of *aac2∆*. Meiotic products of these diploids that lacked chromosomal *FZO1* and *AAC2* were tested for the ability to lose the *FZO1*-carrying plasmid. For eight of 12 isolates tested, Mendelian segregation of a single *sfa* allele appeared likely.

To identify alterations that promote mtDNA maintenance by cells lacking *FZO1*, we turned to bulk segregant analysis (Michelmore *et al.* 1991). From seven of eight *fzo1∆/FZO1aac2∆/AAC2 sfa/SFA* diploids, those haploid segregants proliferating very slowly after loss of the *FZO1* expressing plasmid were differentiated from those that were clearly suppressed and more rapidly proliferating. Suppressor-containing isolates were pooled, and suppressor-lacking isolates were placed into a separate pool. Next, we performed next-generation sequencing of genomic DNA and computationally identified mutations private only to the *sfa* allele-containing pools.

### Mutation of transcription factors of the PDR pathway promotes mtDNA maintenance of cells lacking both *FZO1* and *AAC2*

Surprisingly, all *sfa* mutations sequenced exhibited single amino acid changes to transcription factors Pdr1 or Pdr3, proteins responsible for activation of the PDR pathway and consequent resistance to a panoply of drugs (Balzi *et al.* 1994; Katzmann *et al.* 1994; Prasad and Goffeau 2012). Six missense *PDR1* mutations were identified, along with a single *PDR3* mutation. Table 1 provides the location of each mutation within either Pdr1p or Pdr3p. Interestingly, two independently isolated L868S mutations within Pdr1p suppress the mtDNA loss of *fzo1∆ aac2∆* cells. Therefore, we focused most of our subsequent efforts on the *PDR1-249* allele, which carries this specific amino acid change.

**Table 1:** Alleles of *PDR1* and *PDR3* rescue proliferation of cells lacking both *FZO1* and *AAC2*

Allele	Chromosome Position	Gene	Reference Nucleotide	Mutant Nucleotide	Protein Change
*sfa14*	chrVII:471509	*PDR1*	C	A	D264Y
*sfa142*	chrVII:469696	*PDR1*	A	G	L868S
*sfa159*	chrII:218307	*PDR3*	A	G	R280G
*sfa196*	chrVII:470681	*PDR1*	T	C	K540E
*sfa233*	chrVII:469957	*PDR1*	A	G	V781A
*sfa248*	chrVII:469709	*PDR1*	A	G	Y864H
*sfa249*	chrVII:469696	*PDR1*	A	G	L868S

**Figure 2:**
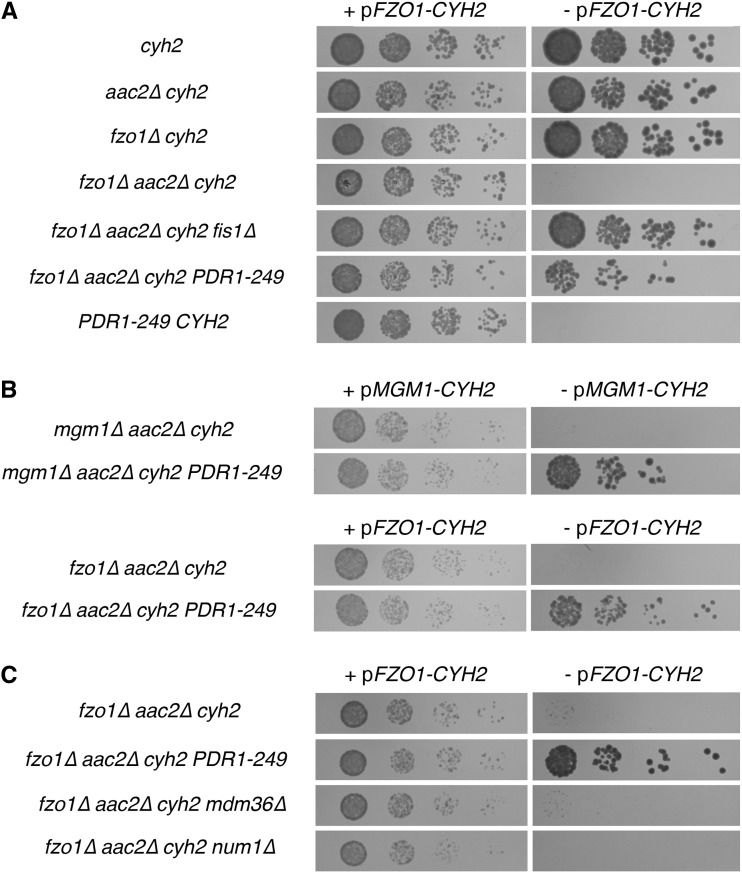
Mutation of *PDR1* promotes maintenance of mitochondrial DNA (mtDNA) by cells lacking mitochondrial fusion. (A) The *PDR1-249* allele allows fusion-incompetent mitochondria to maintain mtDNA in petite-negative cells lacking the ATP/ADP antiporter Aac2p. Strains CDD696 (*cyh2*), CDD698 (*aac2∆ cyh2*), CDD132 (*fzo1∆ cyh2*), CDD71 (*fzo1∆ aac2∆ cyh2*), CDD687 (*fzo1∆ aac2∆ cyh2 fis1∆*), CDD664 (*fzo1∆ aac2∆ cyh2 PDR1-249*), and CDD658 (*PDR1-249 CYH2*), all containing counterselectable plasmid b19 (p*FZO1-CYH2*), were cultured overnight in YEPD medium at 30° then serially diluted and spotted to YEPD medium either lacking (+ p*FZO1-CYH2*) or containing (− p*FZO1-CYH2*) 10 µg/mL CHX and incubated for 1 or 3 d, respectively. (B) Suppression of mtDNA loss from mutants that cannot fuse mitochondria is not limited to cells lacking *FZO1*. Strains CDD717 (*mgm1∆ aac2∆*), CDD716 (*mgm1∆ aac2∆ PDR1-249*), CDD71 (*fzo1∆ aac2∆*), and CDD664 (*fzo1∆ aac2∆ PDR1-249*), all also containing a chromosomal *cyh2* allele and plasmids complementing the deletion of a mitochondrial fusion component, were treated as in (A) to allow for the presence or to select for the absence of plasmid b86 (p*MGM1-CYH2*) or of plasmid b19 (p*FZO1-CYH2*), as indicated. (C) Deletion of *MDM36* or *NUM1* does not permit mtDNA maintenance of mtDNA by cells lacking *FZO1*. Strains CDD71 (*fzo1∆ aac2∆*), CDD664 (*fzo1∆ aac2∆ PDR1-249*), CDD714 (*fzo1∆ aac2∆ mdm36∆*), and CDD750 (*fzo1∆ aac2∆ num1∆*), also carrying a chromosomal *cyh2* mutation and plasmid b19 (*pFZO1-CYH2*), were treated as in (A).

Suppression of mtDNA loss from *fzo1∆ aac2∆* cells by the *PDR1-249* allele was compared to the suppression mediated by deletion of a gene encoding a known mitochondrial division component for *S. cerevisiae*, *FIS1*. In spot dilution assays, the *PDR1-249* allele permitted a similar level of proliferation by *fzo1∆ aac2∆* cells when compared with *fzo1∆ aac2∆ fis1∆* cells (Figure 2A). Because counterselection of the *FZO1*-containing plasmid was based upon CHX treatment, and CHX is a known substrate of the PDR pathway (Saunders and Rank 1982), we verified that our isolation of *PDR1* and *PDR3* alleles as suppressors of *fzo1∆ aac2∆* mtDNA loss was not an artifact of our selection scheme. Indeed, we found that the *PDR1-249* allele does not cause resistance to the concentration of CHX used to counterselect for *FZO1*-containing plasmid, and PCR confirmed absence of *FZO1* from *fzo1∆ aac2∆* cells isolated following counterselection against the p*FZO1-CYH2* plasmid (Figure S1). In addition, when CHX-based counterselection is applied to select for *FZO1* loss, subsequent removal of CHX does not affect the further proliferation of *fzo1∆ aac2∆ PDR1-249* cells, indicating that any CHX-driven modulation of the PDR pathway (Thakur *et al.* 2008) is not relevant to the genetic interaction that we have uncovered (Figure S2). Moreover, we were able to easily isolate *fzo1∆ aac2∆ PDR1-249 CYH2* colonies that had lost the *FZO1*-containing plasmid without the use of counterselection agent CHX, and *FZO1* loss from these isolates was additionally confirmed by PCR (C. D. Dunn, unpublished data), indicating that neither CHX nor a chromosomal *cyh2* allele is required for the *PDR1-249* allele to suppress mtDNA loss from *fzo1∆* cells. We note that after incubation beyond three days at 30°, for *aac2∆* cells lacking *FZO1* and wild-type for *PDR1* and *PDR3*, smaller, slowly proliferating colonies became apparent. Mating these colonies to a *ρ*^-^ tester strain confirmed the presence of mtDNA (Figure S3), suggesting that some fraction of *fzo1∆ aac2∆* cells are able to maintain mtDNA on fermentable medium, even in the absence of a known suppressor mutation. This low level of mtDNA maintenance seems to be a consequence of *AAC2* deletion; cells lacking *FZO1* yet expressing *AAC2* do not maintain mtDNA on fermentable YEPD medium, as further explored herein.

To further characterize the effects of *PDR1* mutation, we tested the ability of the *PDR1-249* allele to suppress mtDNA loss on nonfermentable medium from cells expressing *AAC2* and deficient for mitochondrial fusion. *fzo1∆ cyh2* p*FZO1-CYH2* cells containing or lacking the *PDR1-249* allele were allowed to lose the *FZO1*-encoding plasmid in YEPD medium then subjected to plasmid counterselection on YEPGE medium containing CHX. *fzo1∆ cyh2PDR1-249* cells, upon very extended incubation, did exhibit increased proliferation on YEPGE medium compared to *fzo1∆ cyh2* cells (C. D. Dunn, unpublished data). However analysis by PCR using primers amplifying *FZO1* coding sequence (Figure S1) or a test of tryptophan prototrophy relying upon the *TRP1* gene on the *FZO1*-containing plasmid (C. D. Dunn, unpublished data) demonstrated that *FZO1* was still present. Therefore, in contrast with *fzo1∆ aac2∆ PDR1-249* cells, *fzo1∆ PDR1-249* cells tested upon YEPGE medium did not maintain mtDNA.

We performed additional experiments in YEPD medium testing the potential requirement for *AAC2* deletion in the ability of *fzo1∆ PDR1-249* mutants to maintain mtDNA. We transferred *fzo1∆ fis1∆* strain CDD688 and *fzo1∆ PDR1-249* strain CDD670, both strains containing plasmid-borne *FZO1*, expressing *AAC2*, and harboring a chromosomal *cyh2* mutation, to YEPD liquid medium overnight. Overnight culture in YEPD medium allowed some cells to lose the *FZO1*-containing plasmid during cell division. Subsequently, we plated cells to YEPD medium containing 10 µg/mL CHX, upon which only cells lacking the *FZO1*-encoding plasmid would be expected to survive. Although 75% of individual *fzo1∆ fis1∆* colonies (n = 394) had maintained mtDNA, as determined by replica-plating to solid YEPGE medium, not a single *fzo1∆ PDR1-249* colony exhibited proliferation after replica-plating to YEPGE (n = 446), suggesting a very high rate of mtDNA loss. Mating to *ρ*^-^ strain CDD619, followed by replica-plating of resultant diploids to YEPGE medium confirmed the lack of mtDNA within these *fzo1∆ PDR1-249* colonies. Failure of the *PDR1-249* allele to allow *fzo1∆* mutants expressing *AAC2* to keep mtDNA on both fermentable and non-fermentable medium suggests that absence of *AAC2* may be an important determinant of whether *PDR1* and *PDR3* mutations can allow fusion-defective mitochondria to keep mtDNA.

We asked whether the suppression of mtDNA loss by *PDR1-249* was unique to cells lacking *FZO1* or whether the loss of mtDNA prompted by other mutations blocking mitochondrial fusion similarly could be suppressed by *PDR1* mutation in the background of an *aac2∆* mutant. *MGM1* is required for mitochondrial fusion (Sesaki *et al.* 2003; Wong *et al.* 2003), and mtDNA is, as in *fzo1∆* cells, lost upon Mgm1p inactivation. We placed the *PDR1-249* allele into the context of a *mgm1∆ aac2∆* cell carrying a counterselectable plasmid encoding *MGM1*. Like *fzo1∆ aac2∆* cells, *mgm1∆ aac2∆* cells proliferate in the presence of the *PDR1-249* allele (Figure 2B), demonstrating that the suppression mediated by PDR activation is not specific to the *fzo1∆* mutant.

### Disruption of the Mitochondrial-Endoplasmic Reticulum-Cortex Anchor does not suppress the defective proliferation of petite-negative cells lacking mitochondrial fusion

The Mitochondrial-Endoplasmic Reticulum-Cortex Anchor, or MECA, tethers mitochondria to the plasma membrane of the *S. cerevisiae* mother cell during cytokinesis (Lackner *et al.* 2013). Abrogation of the MECA leads to several phenotypes associated with a mitochondrial division defect, including a highly networked mitochondrial morphology (Cerveny *et al.* 2007; Hammermeister *et al.* 2010). The role of MECA in mitochondrial division is thought to be indirect (Lackner *et al.* 2013), resting upon its ability to tether mitochondria to the plasma membrane and to thereby provide tension to the mitochondrial tubule (Klecker *et al.* 2013). Deletion of MECA component Num1p does not permit mtDNA maintenance by *fzo1∆* cells on YEPGE (Cerveny *et al.* 2007). However, we considered that our selection scheme on fermentable medium might be a more sensitive scheme for uncovering mutants playing a role in mitochondrial division. Therefore, we also tested on YEPD medium whether deletion of the MECA subunits Mdm36p or Num1p would allow proliferation of *fzo1∆ aac2∆* cells. However, proliferation of neither *fzo1∆ aac2∆ mdm36∆* cells nor *fzo1∆ aac2∆ num1∆* mutants could surpass that of *fzo1∆ aac2∆* cells (Figure 2C), suggesting that our selection scheme might not have isolated mutations only providing a partial loss of mitochondrial fission.

### Suppression of mtDNA loss from cells lacking *FZO1* and *AAC2* is associated with dominant activation of the PDR pathway

**Figure 3:**
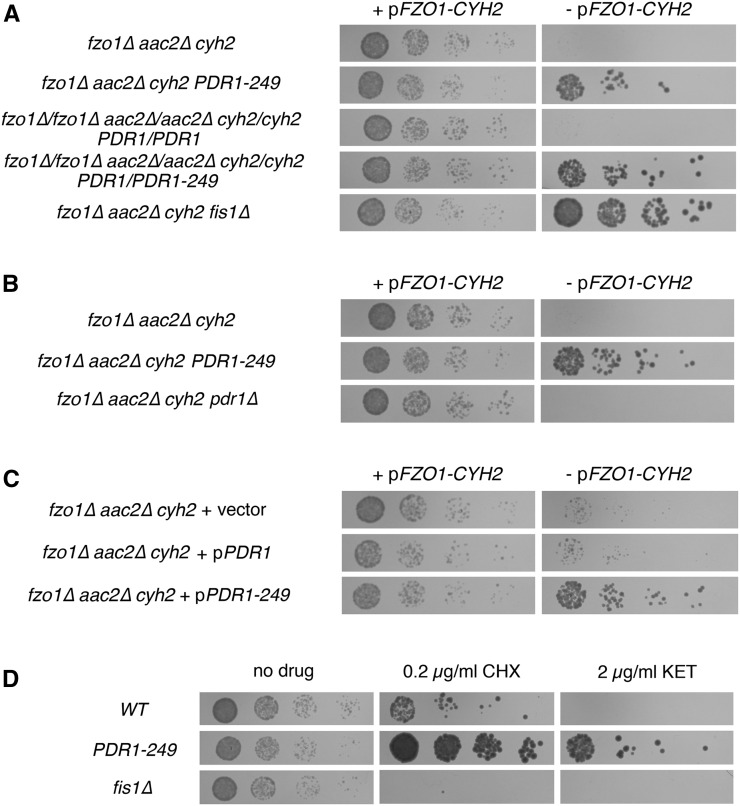
The *PDR1-249* allele dominantly suppresses mitochondrial DNA (mtDNA) loss from mutants unable to fuse mitochondria and activates the PDR pathway. (A) *PDR1-249* dominantly allows mtDNA maintenance in cells lacking *FZO1*. Strains CDD71 (*fzo1∆ aac2∆*), CDD664 (*fzo1∆ aac2∆ PDR1-249*), CDD703 (*fzo1∆/fzo1∆ aac2∆/aac2∆ PDR1/PDR1*), CDD704 (*fzo1∆/fzo1∆ aac2∆/aac2∆ PDR1-249/PDR1*), and CDD687 (*fzo1∆ aac2∆ fis1∆*), all also homozygous for the *cyh2* allele and carrying plasmid b19 (p*FZO1-CYH2*), were treated as in Figure 2A. (B) Deletion of *PDR1* does not suppress mtDNA loss from cells lacking mitochondrial fusion. Strains CDD71 (*fzo1∆ aac2∆*), CDD664 (*fzo1∆ aac2∆ PDR1-249*), and CDD672 (*fzo1∆ aac2∆ pdr1∆*), all also mutated at the *CYH2* locus and carrying plasmid b19 (p*FZO1-CYH2*), were treated as in Figure 2A. (C) Plasmid-borne *PDR1-249* can suppress mtDNA loss from *fzo1∆* cells. *fzo1∆ aac2∆* strain CDD67, also harboring a chromosomal *cyh2* mutation and plasmid b19 (p*FZO1-CYH2*), was transformed with empty vector pRS313 (Sikorski and Hieter 1989), plasmid b60 (p*PDR1*), or plasmid b65 (p*PDR1-249*). Transformants were cultured overnight in SC-Trp medium, then again overnight in SMM-His medium before an assay for proliferation following *FZO1* loss as in Figure 2A. (D) The *PDR1-249* allele activates the PDR pathway. Strains CDD642 (*WT*), CDD658 (*PDR1-249*), and CDD692 (*fis1∆*) were cultured overnight in YEPD at 30°, then serially diluted and spotted to YEPD medium alone (no drug) for 1 d at 30°, YEPD containing 0.2 µg/mL CHX for 3 d, or YEPD containing 2 µg/mL ketoconazole for 3 d.

Because the L868S mutation of Pdr1p was independently isolated twice during our initial selection, we inferred that our suppressor alleles may be dominant; if suppressor mutations in the nonessential *PDR1* gene were recessive, loss-of-function alleles, it would be extremely unlikely to independently isolate the same amino acid change twice. To test whether *PDR1-249* could dominantly suppress mtDNA loss from cells lacking *FZO1* and *AAC2*, we generated diploid cells homozygous for *fzo1∆* and *aac2∆* mutations and either heterozygous for the *PDR1-249* allele or homozygous for the *WT PDR1* allele. Indeed, the presence of a single *PDR1-249* allele allowed maintenance of mtDNA in *fzo1∆/fzo1∆ aac2∆/aac2∆* cells (Figure 3A). Suggesting that *PDR1-249* dominance does not result from haploinsufficiency, we found that deletion of *PDR1* does not suppress mtDNA loss from *fzo1∆ aac2∆* cells (Figure 3B). Furthermore, a plasmid carrying the *PDR1-249* allele was able to dominantly suppress the loss of mtDNA from cells lacking both *FZO1* and *AAC2* (Figure 3C), and other isolated *PDR1* alleles similarly provide dominant suppression of the *fzo1∆ aac2∆* synthetic fitness defect (Figure S4).

To further investigate whether *PDR1-249* is a gain-of-function allele activating the PDR pathway, we plated *PDR1-249* cells and isogenic *WT* cells to YEPD medium containing CHX (Saunders and Rank 1982) or ketoconazole, resistance to which is stimulated by PDR pathway activation (Saunders and Rank 1982; Carvajal *et al.* 1997). Note that the concentration of CHX used in this experiment assaying PDR activation is 20-fold lower than the CHX concentration used for plasmid counterselection purposes. Indeed, *PDR1-249* cells were resistant to both drugs (Figure 3D), signaling activation of the PDR pathway. Cells lacking the mitochondrial division component Fis1p, however, were not resistant to these agents, and even exhibited increased sensitivity to CHX, demonstrating that not all mutations permitting mtDNA maintenance by *fzo1∆ aac2∆* cells lead to PDR activation.

One previously isolated allele of *PDR1* that stimulated drug resistance, *PDR1-2*, was reported to provide a proliferation defect on non-fermentable medium (Balzi *et al.* 1987). However, unlike the *PDR1-2* allele, the *PDR1-249* mutation in an otherwise *WT* background causes no significant defect in cell proliferation on YEPD or YEPGE at any temperature tested (Figure S5).

### Mitochondrial morphology is minimally affected by PDR activation

**Figure 4:**
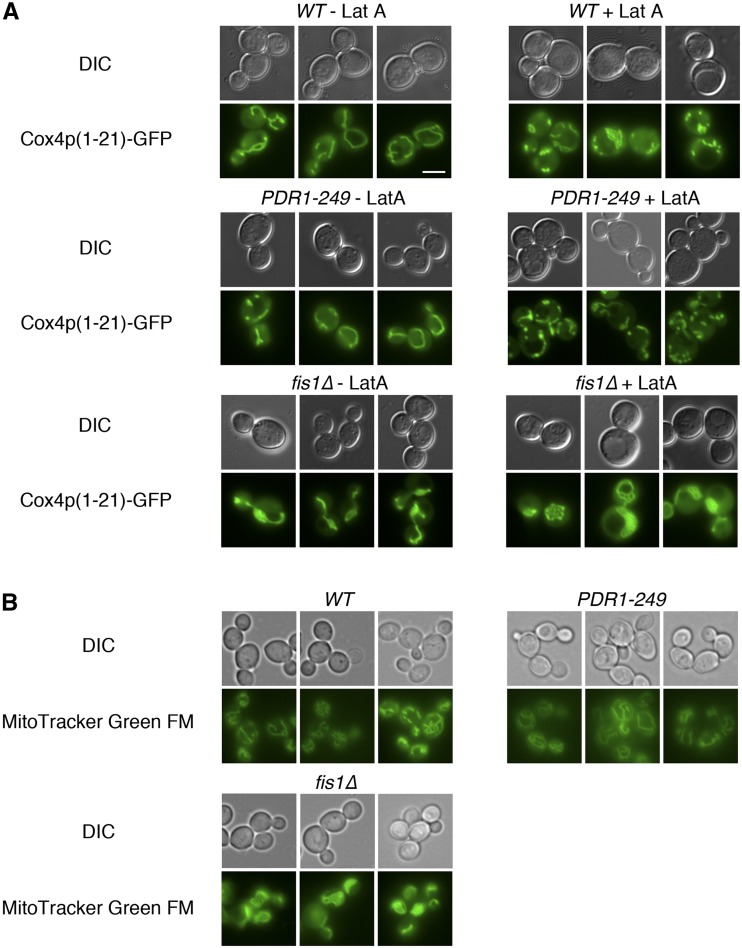
PDR activation by the *PDR1-249* allele does not cause gross changes to mitochondrial shape. (A) Increased mitochondrial fenestration is not apparent in *PDR1-249* cells after disruption of the actin cytoskeleton. Strains CDD642 (*wild type*; *WT*), CDD658 (*PDR1-249*), and CDD692 (*fis1∆*) were transformed with plasmid pHS12 encoding Cox4p(1-21)-GFP (Sesaki and Jensen 1999). During the logarithmic phase of culture in SD-Leu medium, cells were treated with 10 µM latrunculin A (+LatA) or treated with an equal volume of dimethyl sulfoxide vehicle (−LatA) for 1 hr at 30° (Hammermeister *et al.* 2010), followed by fluorescence microscopy. (B) The *PDR1-249* does not lead to morphology changes indicative of a mitochondrial division defect upon non-fermentable medium. Strains CDD642 (*WT*), CDD658 (*PDR1-249*), and CDD692 (*fis1∆*) were cultured in YEPGE medium at the logarithmic phase of proliferation, and mitochondrial morphology was visualized following staining with 500 nM MitoTracker Green FM. Bar, 5 µm.

Because genetic evidence suggested that PDR activation might inhibit mitochondrial division, we examined the mitochondrial morphology of *PDR1-249* cells by fluorescence microscopy after transformation with GFP targeted to mitochondria by the Cox4 presequence (Sesaki and Jensen 1999), but initial observation indicated that there was minimal, if any difference in mitochondrial morphology between *WT* and *PDR1-249* cells. Disruption of the actin cytoskeleton causes fragmentation of all but the most highly networked mitochondrial tubules, better revealing fenestrated mitochondria that might result from a decrease in the rate of mitochondrial division (Bleazard *et al.* 1999). However, use of latrunculin A to disrupt the actin cytoskeleton and mitochondrial structure (Jensen *et al.* 2000; Hammermeister *et al.* 2010) did not reveal an apparent increase in mitochondrial fenestration when comparing *WT* cells to *PDR1-249* cells, while *fis1∆* networks were readily seen both before and after treatment with latrunculin A (Figure 4A). Quantification of cells containing mitochondrial networks after the addition of latrunculin A also demonstrated that PDR activation does not increase the interconnectedness of mitochondria (Figure S6). Examination of *PDR1-249* cells cultured in nonfermentable YEPGE medium by MitoTracker Green FM staining also did not uncover any mitochondrial morphology defect (Figure 4B).

We next examined the morphology of *fzo1∆* cells carrying the *PDR1-249* allele to examine the effect of PDR activation when mitochondrial fusion is blocked. For cells expressing *AAC2*, we compared mutants lacking mtDNA so that *fzo1∆* cells with a wild-type *PDR1* allele might be used as a control. However, this approach failed to uncover a difference between the mitochondrial morphology of *fzo1∆ ρ^0^* and *fzo1∆ PDR1-249 ρ^0^* cells (Figure S7), although some *fzo1∆ fis1∆ ρ^0^* cells did exhibit networked mitochondria. We also characterized the mitochondrial shape of *fzo1∆ aac2∆ PDR1-249 ρ^+^* cells and found that mitochondria were typically clustered at a single location and were not tubular. This mitochondrial morphology differed from that of *fzo1∆ aac2∆ fis1∆ ρ^+^* cells, which exhibited a more elongated mitochondrial shape.

### Altered mitochondrial genome distribution, increased mtDNA abundance, or overexpression of a mitochondrial protein import receptor is unlikely to allow maintenance of mtDNA upon a block to mitochondrial fusion

**Figure 5:**
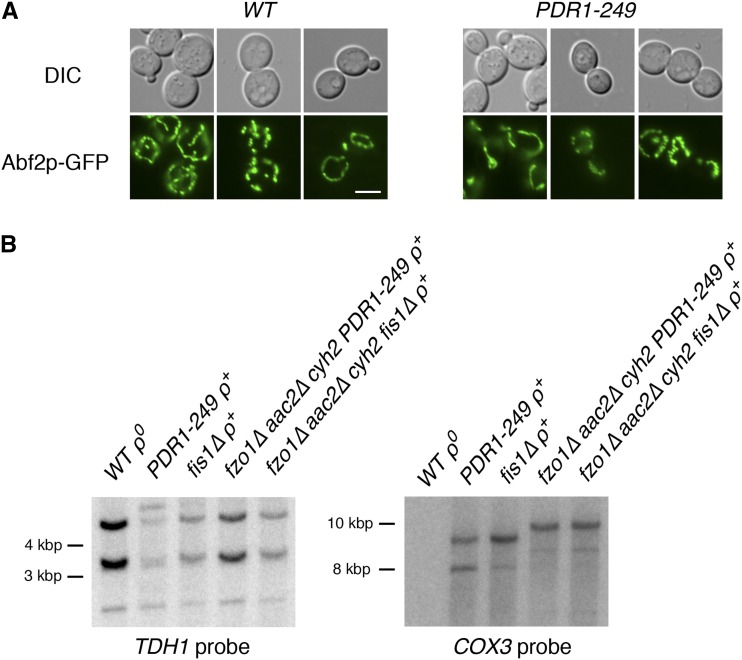
PDR activation by the *PDR1-249* allele does not cause gross changes to mitochondrial nucleoid distribution or mitochondrial DNA (mtDNA) abundance. (A) The distribution of mitochondrial nucleoids between mother and bud cells does not appear abnormal in *PDR1-249* cells. Strains CDD642 (*WT*) and CDD658 (*PDR1-249*) were transformed with plasmid pM390, encoding GFP-tagged Abf2p. Mitochondrial morphology was visualized in SD-Leu medium during the logarithmic phase of culture growth. Bar, 5 µm. (B) Mitochondrial DNA is not amplified by *PDR1-249* cells. Equal amounts of genomic DNA from an isolate of strain CDD642 lacking mtDNA after treatment with ethidium bromide (*WT ρ^0^*), CDD658 (*PDR1-249*), CDD692 (*fis1∆*), and strains CDD664 (*fzo1∆ aac2∆ PDR1-249*) and CDD687 (*fzo1∆ aac2∆ fis1∆*), both lacking plasmid b19 (p*FZO1 -CYH2)* following CHX counterselection, were cut with *Eco*RV and Southern blotting was performed using probes recognizing nuclear gene *TDH1* or mitochondrial gene *COX3*.

Potentially, an increase in the transport of mtDNA to the bud of dividing cells with an activated PDR pathway might promote mtDNA retention when mitochondrial fusion is perturbed. To visualize mtDNA in cells with a hyperactive PDR pathway, we expressed a fusion protein consisting of the nucleoid protein Abf2 linked to GFP (Okamoto *et al.* 1998). Upon examination of nucleoids in live *WT* and *PDR1-249* cells using Abf2p-GFP, no obvious change in the distribution of nucleoids between mother and bud was apparent (Figure 5A), and similar results were obtained by DAPI staining of nucleoids in live cells (C. D. Dunn, unpublished data). Nucleoids did appear to be less well defined in *PDR1-249* cells, with reduced delineation between mtDNA puncta; however, the etiology of any minor effect on internucleoid distance induced by PDR activation remains to be determined. In any case, because mtDNA nucleoids did not seem to be increased in the bud of *PDR1-249* cells compared with *WT* cells, more rapid transmission of mtDNA to the growing bud at the cost of maternal mtDNA retention is unlikely to be the mechanism by which *fzo1∆* cells maintain mtDNA when PDR is activated.

Alternatively, a drastic increase in mtDNA abundance caused by PDR activation might counterbalance an increased rate of mtDNA loss from mitochondria unable to fuse. We examined mtDNA levels in *PDR1-249* cells and in *fzo1∆ aac2∆ PDR1-249* cells, but we found no increase in mtDNA compared to *fzo1∆ aac2∆ fis1∆* cells, in which mtDNA is instead maintained due to a blockade of mitochondrial fission, or to *fis1∆* cells (Figure 5B). Therefore, mtDNA amplification is also unlikely to be the mechanism by which PDR activation protects cells with defective mitochondrial fusion from mtDNA loss. Interestingly, we found a potential rearrangement of mtDNA in *fzo1∆ aac2∆ PDR1-249* and *fzo1∆ aac2∆ fis1∆* cells, as reported by a change in the mobility of an mtDNA restriction fragment containing *COX3*. In this regard, it is noteworthy that mtDNA instability is a well-known consequence of mutating the ANT1 ATP/ADP antiporter in mammals (Esposito *et al.* 1999; Kaukonen *et al.* 2000). In any case, we have confirmed that the suppressive effect of PDR activation on mtDNA loss from mitochondrial fusion mutants is not dependent upon any specific mtDNA structure found in the strain used for our initial selection; we regenerated a *fzo1∆ aac2∆ PDR1-249* strain whose parental mtDNA is inherited from a wild-type strain of the same background (BMA64-1A) and then recapitulated our results demonstrating that PDR activation suppresses mtDNA disappearance from *aac2∆* cells after loss of *FZO1* (C. D. Dunn, unpublished data).

PDR pathway activation by *PDR3* overproduction increases the levels of Tom71p, a receptor found on the mitochondrial OM and involved in the import of nucleus-encoded mitochondrial proteins (Koh *et al.* 2001). Tom71p also recruits the F-box protein Mfb1, a polypeptide required for normal mitochondrial morphology and tubule size, to the mitochondrial surface (Kondo-Okamoto *et al.* 2008). Therefore, we tested whether Tom71p was required for the suppression of mtDNA loss from *fzo1∆* cells by PDR pathway up-regulation. However, deletion of the *TOM71* gene had no effect on the proliferation of *fzo1∆ aac2∆ PDR1-249* cells (Figure S8).

## Discussion

We performed a genetic selection, taking advantage of the petite-negative phenotype, the goal of which was to identify new genes and pathways involved in mitochondrial dynamics. After next-generation sequencing of suppressor-containing genomes, we found that activation of the PDR pathway can, under certain circumstances, allow cells deficient in mitochondrial fusion to keep mtDNA. We note that next-generation sequencing of segregant pools greatly facilitated the rapid identification of the dominant alleles described here, because identification of dominant mutations typically requires either arduous construction of a genomic library or, alternatively, painstaking work to map the relevant variant (Forsburg 2001).

Interestingly, mutation of the *FZO1* gene in *S. cerevisiae* has previously been reported to activate the PDR pathway via the Pdr3p transcription factor. However, this effect seems to result from the loss of mtDNA precipitated by blockade of mitochondrial fusion and is likely not related to mitochondrial dynamics *per se* (Hallstrom and Moye-Rowley 2000). Studies of pathogenic yeast also demonstrated a link between the absence of mtDNA and PDR pathway activation (Sanglard *et al.* 2001; Ferrari *et al.* 2011). Although PDR activation prevents, rather than results from, mtDNA loss under the circumstances examined in this study, our findings further link the PDR pathway to mitochondrial function.

One possible mechanism by which mtDNA maintenance by fusion-deficient mitochondria is promoted after PDR activation would be a disruption of mitochondria division. Considering our failure to visualize in *PDR1-249* cells any gross changes to mitochondrial structure characteristic of mutations affecting mitochondrial division, a model where PDR activation reduces mitochondrial division seems difficult to countenance. Moreover, mutations providing prominent changes to mitochondrial morphology by indirectly reducing mitochondrial division, such as removal of MECA complex members Num1p or Mdm36p (Dimmer *et al.* 2002; Hammermeister *et al.* 2010; Klecker *et al.* 2013), fail to allow *fzo1∆ aac2∆* mutants to effectively maintain mtDNA. Since the *PDR1-249* allele only suppressed mtDNA loss in the presence of the *aac2∆* allele and not in *AAC2* cells, it is possible that loss of *AAC2* potentiates the effects of PDR activation and, together, these mutations lead to a block to mitochondrial division. Supporting this possibility, removal of *AAC2* affects mitochondrial morphology (Altmann and Westermann 2005), potentially signaling an uncharacterized role for the Aac2 protein in mitochondrial dynamics that is worthy of future study.

Pursuing other possible ways in which PDR activation might allow mtDNA to be maintained by mitochondrial fusion mutants, we also investigated whether PDR activation greatly altered mtDNA distribution or abundance. However, nucleoid distribution between mother and bud appeared normal in *PDR1-249* mutants, and mtDNA levels were not elevated when compared to cells lacking Fis1p, a protein required for mitochondrial fission, suggesting that amplification of mtDNA or more rapid transfer of mtDNA into the bud does not promote mtDNA retention by the *fzo1∆* mutant. Moreover, overexpression of a PDR-activated protein import receptor, Tom71p, seems irrelevant to the apparent effects of PDR activation on mitochondrial dynamics. It is possible that increased sphingolipid biosynthesis could be relevant to suppression of mtDNA loss from *fzo1∆* mutants by PDR activation, as several enzymes playing a role in sphingolipid biosynthesis are up-regulated by the PDR pathway (Hallstrom *et al.* 2001; Kolaczkowski *et al.* 2004). We sought to perturb sphingolipid biosynthesis by deleting *SUR4*, which plays a role in synthesis of the major sphingolipid forms of *S. cerevisiae* (Oh *et al.* 1997; Dickson 2008). However, we found that *sur4∆* mutants proliferated poorly following germination of haploid spores from a heterozygous diploid (Figure S9), and *fzo1∆ aac2∆ sur4∆* mutants expressing plasmid-borne *FZO1* also exhibit severe proliferation defects on YEPD (C. D. Dunn, unpublished data), confounding immediate analysis of any role for up-regulated sphingolipid biosynthesis downstream of PDR activation.

It has been suggested that screens and selections for new components playing a direct role in mitochondrial division might be saturated (Lackner and Nunnari 2009). Have, in fact, all of the proteins directly taking part in the mitochondrial division reaction in *S. cerevisiae* been identified? Before initiating this study, we noted that numerous proteins involved in membrane curvature and endocytosis at the plasma membrane, such as the BAR domain containing proteins Rvs161 and Rvs167, are required for proliferation on non-fermentable media in at least one yeast background (Ren *et al.* 2006), suggesting potential functionality at mitochondria. Our selection design would likely have discovered any substantial role for such endocytosis regulators in mitochondrial scission, yet no mutations in genes encoding endocytosis components were identified.

We had also hoped that our selection might uncover machinery used to divide the mitochondrial IM, as no proteins have yet been identified that demonstrably play a direct role in the mitochondrial division reaction at this location (Westermann 2010). In fact, exciting new data indicate that some proteins can participate in both oxidative phosphorylation and mitochondrial protein import (Gebert *et al.* 2011), permitting speculation that other IM proteins might also play a dual role, functioning in both mitochondrial ATP generation and mitochondrial division. Our selection scheme did not require that mutations affecting mitochondrial division respire, and so we could have conceivably identified such dual-use proteins. However, we did not isolate mutations in any gene playing a direct role in oxidative phosphorylation. Perhaps a partially functional, yet still petite-negative allele of *AAC2* would have been more suitable for this purpose, since complete *AAC2* deletion is synthetically lethal with an absolute block to either the electron transport chain or to the ATP synthase complex (Smith and Thorsness 2008). Specific allele selection may be particularly important in this context: as described above, the full deletion of *AAC2* may have facilitated recovery of mutations impinging upon mtDNA loss from fusion defective mitochondria.

To summarize, our selection scheme uncovered regulatory proteins that genetically interface with mitochondrial dynamics in an intriguing way. Further studies should elucidate the mechanism by which PDR pathway transcriptional targets allows mtDNA maintenance when mitochondrial fusion is lacking. We note that, although rapid mtDNA loss from a mitochondrial fusion mutant seems related to generation of small, fragmented mitochondria, it is not clear why mtDNA is lost from these fragments. PDR activation may prevent mtDNA loss by addressing the most proximal cause of mtDNA loss from fusion-deficient mitochondria rather than impinging in any way upon mitochondrial dynamics. Finally, our work suggests that alternative methodologies not taking advantage of direct selection are likely to be required for identification of new *S. cerevisiae* proteins that play a direct role in mitochondrial division.

## Supplementary Material

Supporting Information
