# Supplementary material for: Activation of the Pleiotropic Drug Resistance Pathway Can Promote Mitochondrial DNA Retention by Fusion-Defective Mitochondria in Saccharomyces cerevisiae
Source: G3 (Bethesda). 2014 May 6;4(7):1247–58. doi: 10.1534/g3.114.010330 (PMC4455774; doi:10.1534/g3.114.010330)
Supplement: Supporting Information [file supp_4_7_1247__index.html]

Activation of the Pleiotropic Drug Resistance Pathway Can Promote Mitochondrial DNA Retention by Fusion-Defective Mitochondria in Saccharomyces cerevisiae — Supporting Information 

# Activation of the Pleiotropic Drug Resistance Pathway Can Promote Mitochondrial DNA Retention by Fusion-Defective Mitochondria in *Saccharomyces cerevisiae*

## Supporting Information for Mutlu *et al.*, 2014

**Files in this Data Supplement:**

- Supporting Information - Figures S1-S9, Tables S1-S2, and File S1 (PDF, 1 MB)
- Figure S1 - The *PDR1-249* allele permits loss of *FZO1* from cells lacking *AAC2* upon YEPD medium, but not from cells expressing *AAC2*. (PDF, 1 MB)
- Figure S2 - The presence of cycloheximide is not required for suppression of mtDNA loss from *fzo1Δ aac2Δ* cells by *PDR1-249*. (PDF, 249 KB)
- Figure S3 - Viable microcolonies of *fzo1Δ aac2Δ* cells contain mtDNA. (PDF, 3 MB)
- Figure S4 - All isolated *PDR1* mutations act as dominant suppressors of the proliferation defect of *fzo1Δ aac2Δ* cells. (PDF, 241 KB)
- Figure S5 - *PDR1-249* cells exhibit minimal proliferation defects on fermentable or non-fermentable medium. (PDF, 301 KB)
- Figure S6 - Quantification of mitochondrial networks following latrunculin A treatment. (PDF, 1 MB)
- Figure S7 - Examination of the morphology of fusion-defective mitochondria. (PDF, 137 KB)
- Figure S8 - *TOM71* is not required for suppression of mtDNA loss from *fzo1Δ aac2Δ* cells by PDR pathway activation. (PDF, 108 KB)
- Figure S9 - Mutants lacking *sur4Δ* derived from the W303 background of *S. cerevisiae* exhibit a significant proliferation defect. (PDF, 230 KB)
- Table S1 - Strain genotypes and construction. (PDF, 441 KB)
- Table S2 - Oligonucleotides used in this study. (PDF, 153 KB)
- File S1 - VarScan output highlighting the relevant mutation in each suppressor-containing genomic DNA pool. (.xlsx, 6 MB)
