# Supplementary material for: Activation of the Pleiotropic Drug Resistance Pathway Can Promote Mitochondrial DNA Retention by Fusion-Defective Mitochondria in Saccharomyces cerevisiae
Source: G3 (Bethesda). 2014 May 6;4(7):1247–58. doi: 10.1534/g3.114.010330 (PMC4455774; doi:10.1534/g3.114.010330)
Supplement: Supporting Information [file supp_g3.114.010330_010330SI.pdf]

G3· Genes | Genomes | Genetics

Activation of the pleiotropic drug resistance pathway can promote mitochondrial DNA retention by fusion-defective mitochondria in *Saccharomyces cerevisiae*

Nebibe Mutlu [1], Görkem Garipler, Emel Akdoğan and Cory D. Dunn*

Department of Molecular Biology and Genetics
Koç University
Sarıyer, İstanbul, 34450
Turkey

[1] Present address: Department of Molecular, Cellular, and Developmental Biology, University of Michigan, Ann Arbor, Michigan, 48109, U.S.A.

* Address all correspondence to: Dr. Cory D. Dunn, Department of Molecular Biology and Genetics, Koç University, Rumelifeneri Yolu, Sarıyer, İstanbul, 34450, Turkey. Tel: +90 (212) 338 1449; Fax: +90 (212) 338 1559;
Email: cdunn@ku.edu.tr

doi:10.1534/g3.114.010330

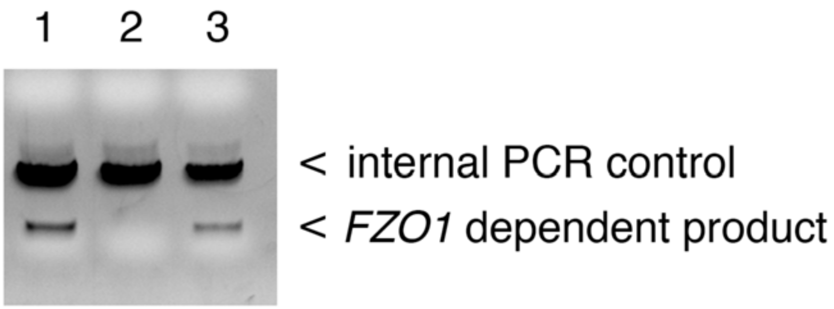

Figure S1 The *PDR1-249* allele permits loss of *FZO1* from cells lacking *AAC2* upon YEPD medium, but not from cells expressing *AAC2*. Genomic DNA was harvested from the following isolates: (1) An example colony from *fzo1Δ cyh2 PDR1-249* strain CDD670, subjected to counter-selection against plasmid b19 (p*FZO1-CYH2*) upon YEPGE containing 3 µg/ml CHX; (2) A colony isolated from *fzo1Δ aac2Δ cyh2 PDR1-249* strain CDD664 lacking plasmid b19 (p*FZO1-CYH2*) following culture on YEPD + 10 µg/ml CHX; and (3) *FZO1* control strain CDD768. A multiplex PCR reaction was performed using primers 54 and 55, amplifying *FIS1* and surrounding sequence (large product), and primers 64 and 65, which amplify a region within the *FZO1* ORF (small product).

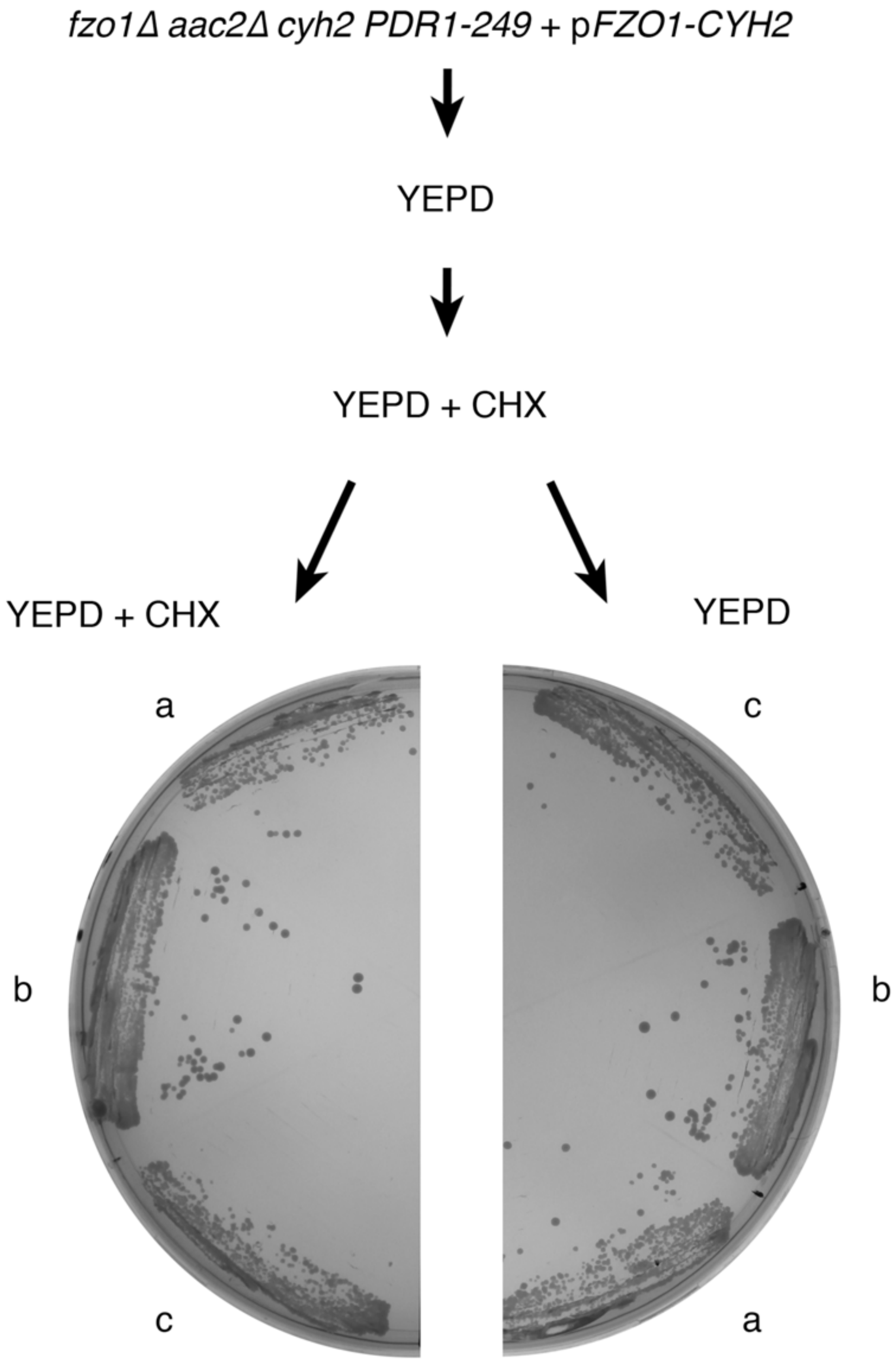

Figure S2 The presence of cycloheximide is not required for suppression of mtDNA loss from *fzo1Δ aac2Δ* cells by *PDR1-249*. Cells from *fzo1Δ aac2Δ PDR1-249* strain CDD664 carrying plasmid b19 (p*FZO1-CYH2)* were struck to YEPD medium for 1 d, followed by culture on YEPD medium containing 10 µg/ml CHX for 3 d to counter-select against plasmid-borne *FZO1*. Next, cells from three viable *fzo1Δ aac2Δ PDR1-249* colonies (denoted a, b, and c) were struck to both YEPD and to YEPD + CHX, then incubated for a further 3 d.

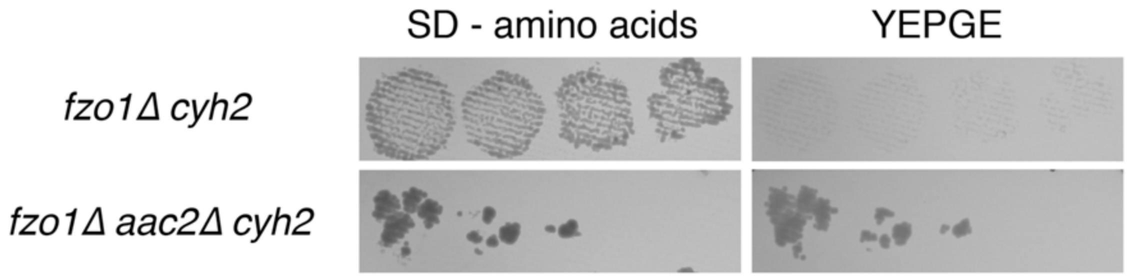

Figure S3 Viable microcolonies of *fzo1Δ aac2Δ* cells contain mtDNA. Microcolonies of strains CDD71 (*fzo1Δ aac2Δ cyh2*) and CDD132 (*fzo1Δ cyh2*) forced to lose plasmid b19 (*pFZO1-CYH2*) in Figure 2A were mated to ρ^- tester strain CDD620. Diploids were replica-plated to SD medium lacking all amino acids in order to demonstrate mating prowess and to YEPGE medium in order to assay the presence of mtDNA. Diploid cells were then incubated for 2 d.

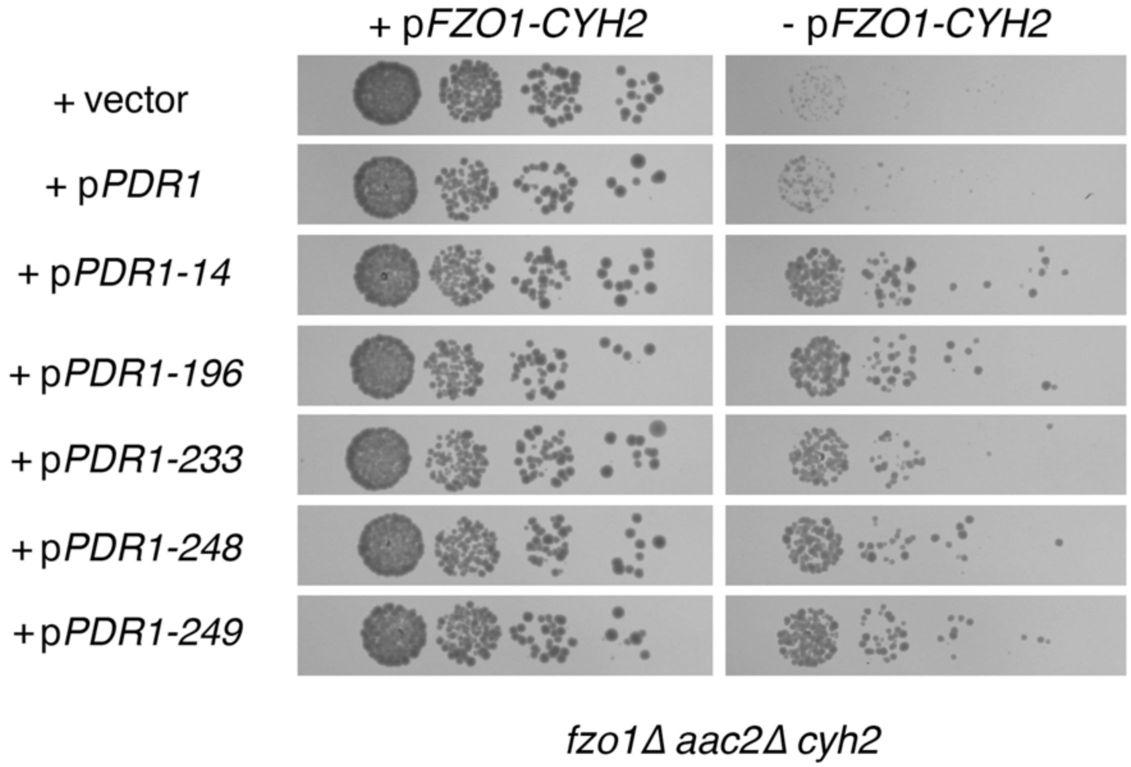

Figure S4 All isolated *PDR1* mutations act as dominant suppressors of the proliferation defect of *fzo1Δ aac2Δ* cells. Strain CDD67 (*fzo1Δ aac2Δ*), also harboring a chromosomal *cyh2* mutation and plasmid b19 (p*FZO1-CYH2*), was transformed with empty vector pRS313, plasmids b60 (p*PDR1*), b61 (p*PDR1-14*), b62 (p*PDR1-196*), b63 (p*PDR1-233*), b64 (p*PDR1-248*), or b65 (p*PDR1-249*). Transformants were treated as in Figure 3C, except that cells not subject to counter-selection using CHX were incubated for 2 d.

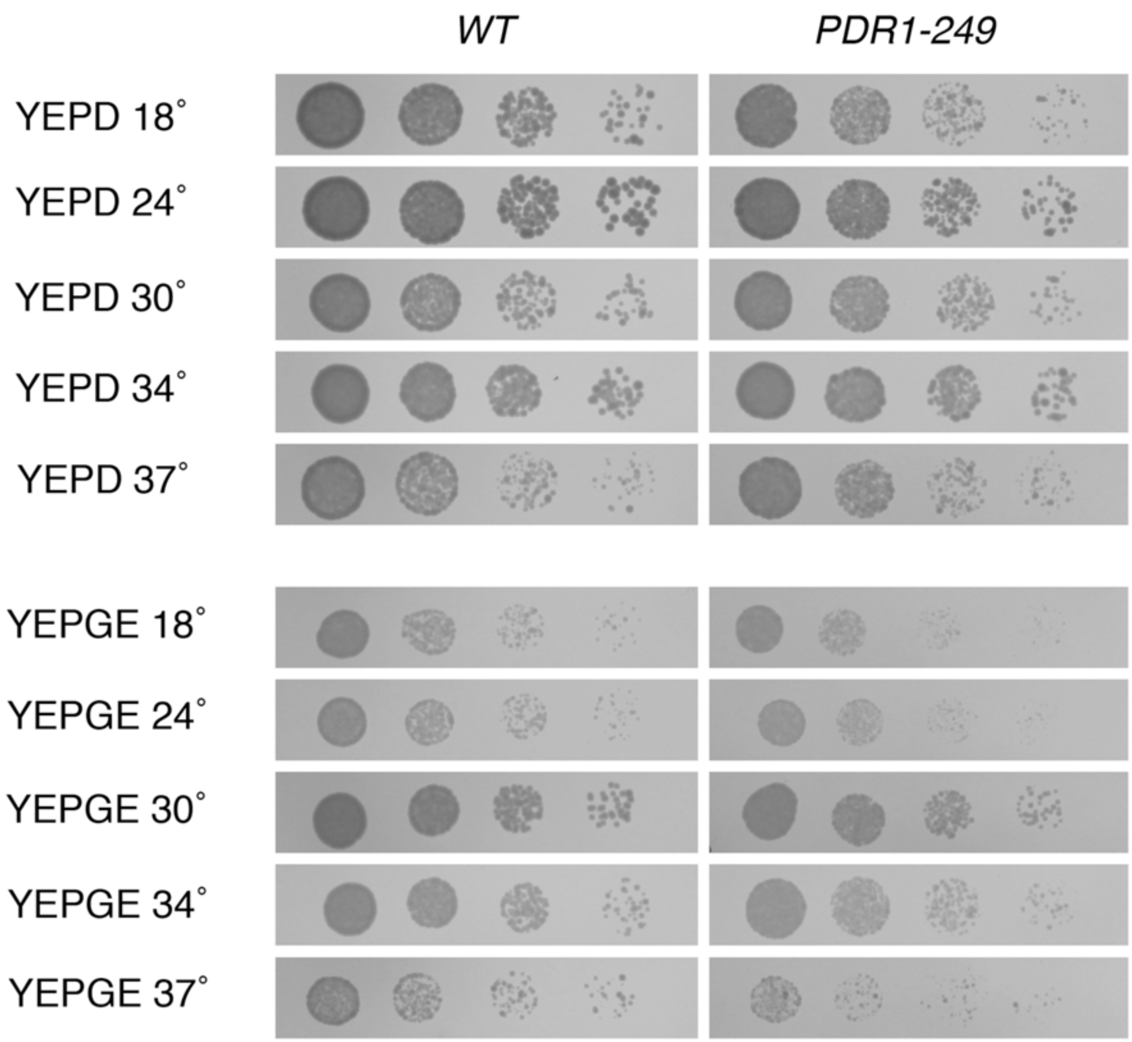

Figure S5 *PDR1-249* cells exhibit minimal proliferation defects on fermentable or non-fermentable medium. Strains CDD642 (*WT*) and CDD658 (*PDR1-249*) were cultured in YEPD overnight at 30˚, then serially diluted and plated at the indicated temperature on either YEPD or YEPGE medium. Cells were incubated for 1 d on YEPD at 30˚, 34˚, and 37˚. Strains were incubated for 2 d on YEPD at 24˚ and on YEPGE at 24˚, 30˚, and 34˚. Cultures were incubated for 3 d on YEPD at 18˚ and on YEPGE at 18˚ and 37˚.

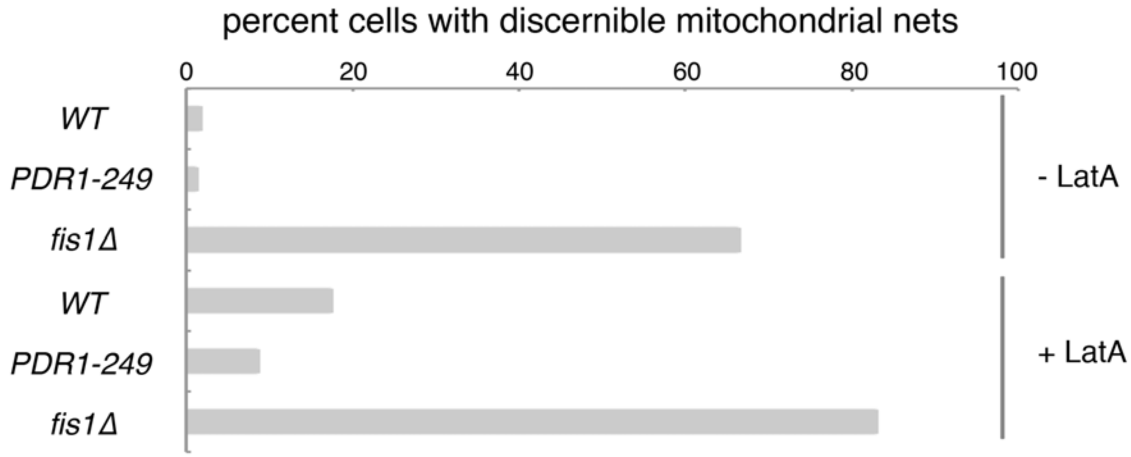

Figure S6 Quantification of mitochondrial networks following latrunculin A treatment. The number of cells containing mitochondrial networks from strains CDD642 (*WT*), CDD658 (*PDR1-249*), and CDD692 (*fis1Δ*), stained with MitoTracker Green FM and shown in Figure 4, was quantified (n>200 cells).

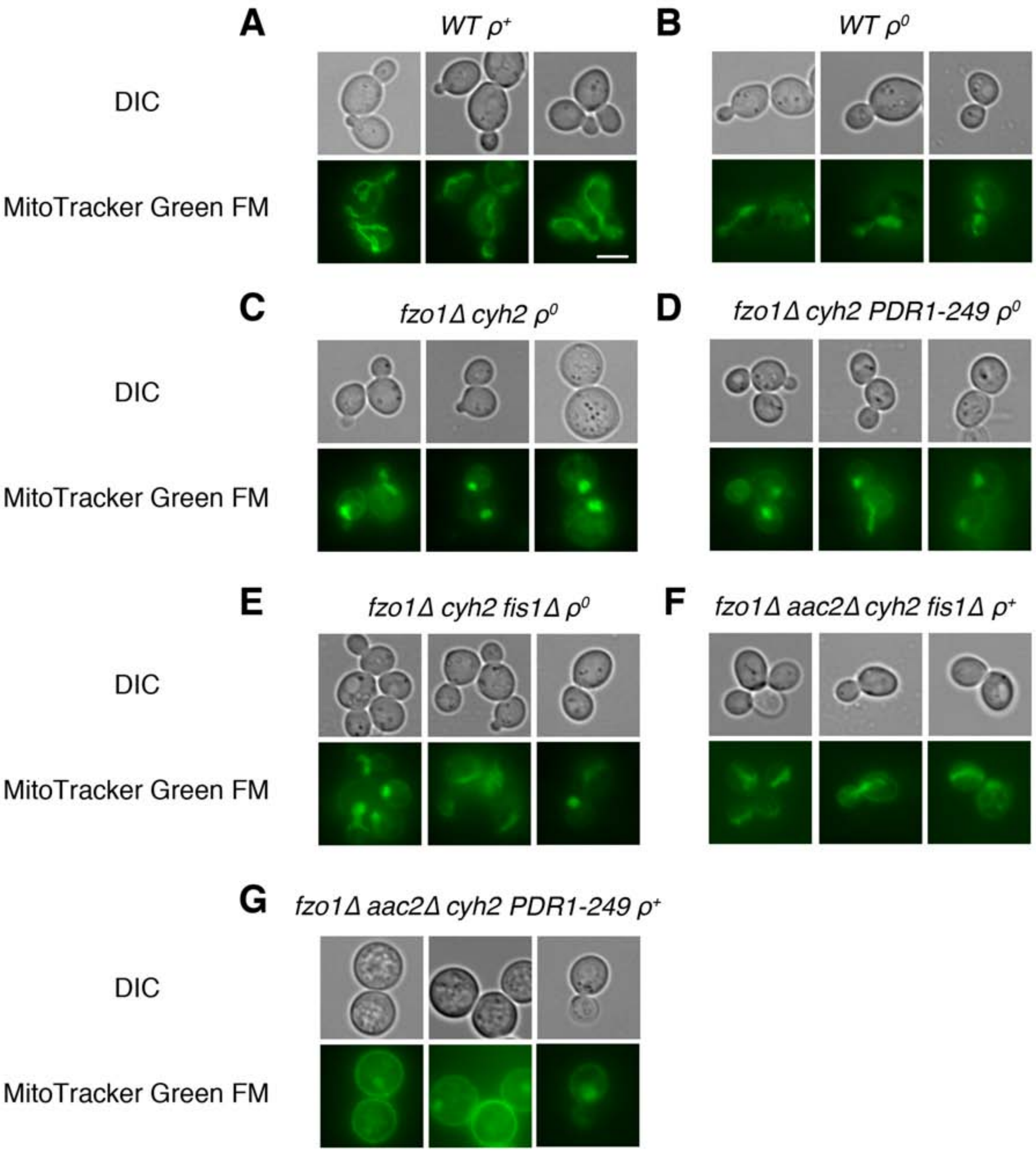

Figure S7 Examination of the morphology of fusion-defective mitochondria. The following strains cultured in YEPD within the logarithmic phase of growth were stained with MitoTracker Green FM and visualized by fluorescence microscopy: (A) CDD642 containing mtDNA (*WT* ρ^+). (B) CDD642 lacking mtDNA following EtBr treatment (*WT* ρ^0). (C) CDD132 lacking mtDNA following counter-selection for p*FZO1-CYH2* plasmid b19 (*fzo1Δ cyh2* ρ^0). (D) CDD670 lacking mtDNA following counter-selection for plasmid b19 (*fzo1Δ cyh2 PDR1-249* ρ^0). (E) CDD688 lacking mtDNA following EtBr treatment and lacking plasmid b19 following counter-selection (*fzo1Δ cyh2Δ fis1Δ* ρ^0). (F) CDD687 lacking plasmid b19 following counter-selection (*fzo1Δ aac2Δ cyh2 fis1Δ* ρ^+). (G) CDD664 lacking plasmid b19 following counter-selection (*fzo1Δ aac2Δ cyh2 fis1Δ* ρ^+). Bar, 5 µm.

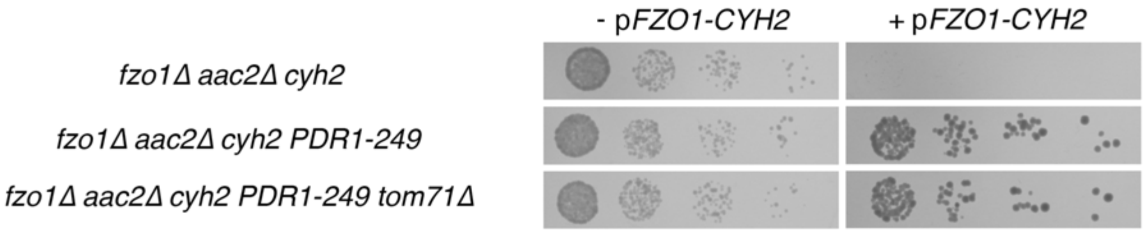

Figure S8 *TOM71* is not required for suppression of mtDNA loss from *fzo1Δ aac2Δ* cells by PDR pathway activation. Strains CDD71 (*fzo1Δ aac2Δ*), CDD664 (*fzo1Δ aac2Δ PDR1-249*), and CDD685 (*fzo1Δ aac2Δ PDR1-249 tom71Δ*), each containing a *cyh2* mutation and plasmid b19 (p*FZO1-CYH2*) were treated as in Figure 2A.

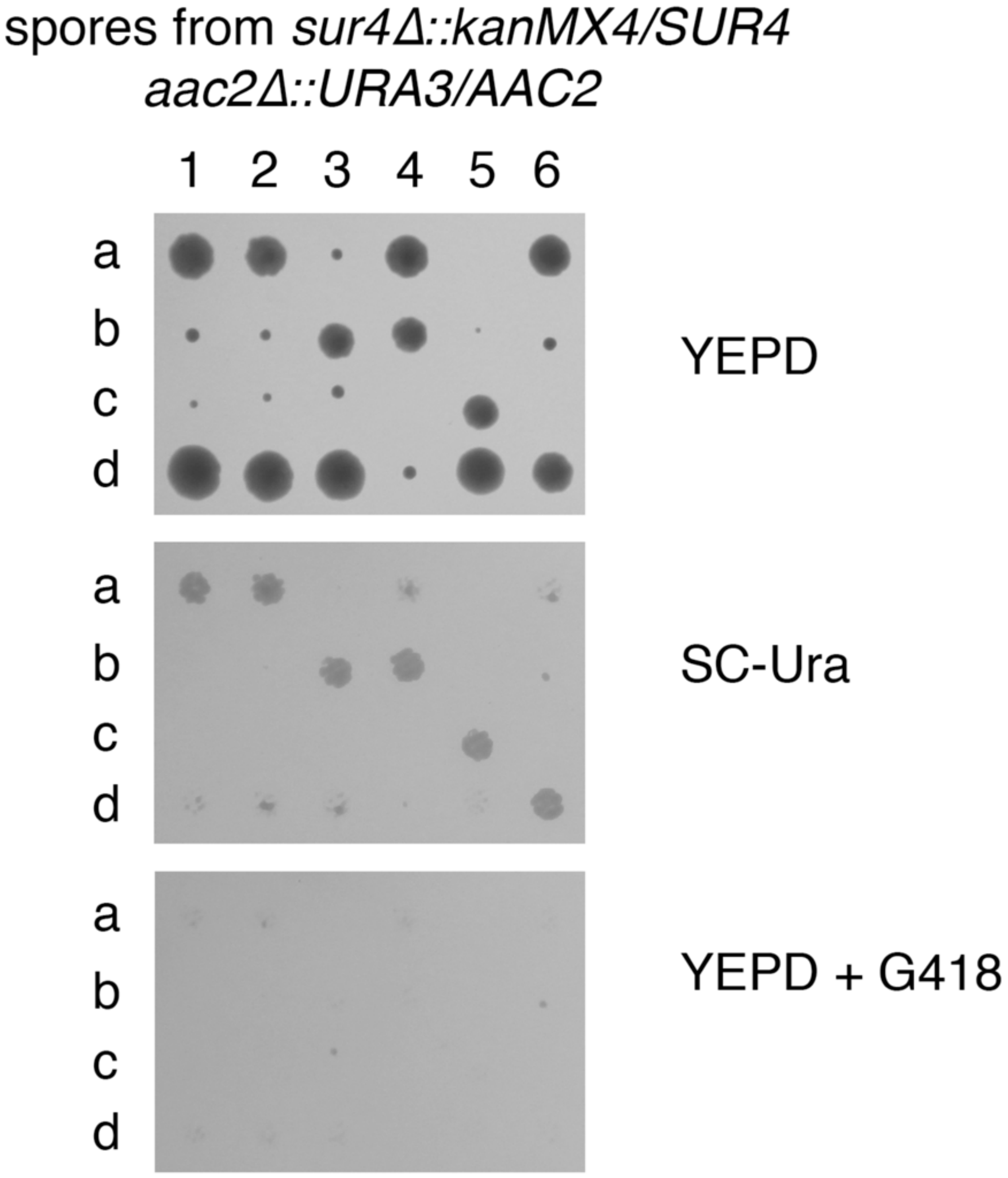

Figure S9 Mutants lacking *sur4Δ* derived from the W303 background of *S. cerevisiae* exhibit a significant proliferation defect. Strain CDD744 (*sur4Δ::kanMX4/SUR4 aac2Δ::URA3/AAC2*) was sporulated, then tetrads were dissected onto YEPD medium and incubated for 4 d. Genotyping was performed by replica plating to SC medium lacking uracil (SC-Ura) or to YEPD containing 200 μg/ml G418, followed by incubation for 1 d.

Table S1 - Strain genotypes and construction.

Strain	Genotype	Source	Parental strain(s)	Method Used
BMA64-1A	*MATa ura3-1 trp1Δ2 ade2-1 leu2-3,112 his3-11,15 can1-100*	Baudin-Baillieu et al. (1997)		
BMA64-1B	*MATα ura3-1 trp1Δ2 ade2-1 leu2-3,112 his3-11,15 can1-100*	Baudin-Baillieu et al. (1997)		
BMA64	*MATα/MATa ura3-1/ura3-1 trp1Δ2/trp1Δ2 ade2-1/ade2-1 leu2-3,112/leu2-3,112 his3-11,15/his3-11,15 can1-100/can1-100*	Baudin-Baillieu et al. (1997)		
RJ98	*MATα lys1*	Robert Jensen		
CDD29	*MATa leu2Δ0 met15Δ0 ura3Δ0 his3Δ1 dnm1Δ::kanMX4*	EUROSCARF Y01489		
CDD36	*MATa leu2Δ0 met15Δ0 ura3Δ0 his3Δ1 mdv1Δ::kanMX4*	EUROSCARF Y01311		
CDD38	*MATa leu2Δ0 met15Δ0 ura3Δ0 his3Δ1 fis1Δ::kanMX4*	EUROSCARF Y01458		
CDD42	*MATa leu2Δ0 met15Δ0 ura3Δ0 his3Δ1 mdm36Δ::kanMX4*	EUROSCARF Y07401		
CDD51	*MATα ura3-1 trp1Δ2 ade2-1 leu2-3,112 his3-11,15 can1-100 cyh2*	Garipler and Dunn (2013)		
CDD53	*MATα/MATa ura3-1/ura3-1 trp1Δ2/trp1Δ2 ade2-1/ade2-1 leu2-3,112/leu2-3,112 his3-11,15/his3-11,15 can1-100/can1-100 cyh2/CYH2*	Garipler and Dunn (2013)		
CDD58	*MATα/MATa ura3-1/ura3-1 trp1Δ2/trp1Δ2 ade2-1/ade2-1 leu2-3,112/leu2-3,112 his3-11,15/his3-11,15 can1-100/can1-100 cyh2/CYH2 aac2Δ::URA3/AAC2*	Garipler and Dunn (2013)		
CDD59	*MATα/MATa ura3-1/ura3-1 trp1Δ2/trp1Δ2 ade2-1/ade2-1 leu2-3,112/leu2-3,112 his3-11,15/his3-11,15 can1-100/can1-100 cyh2/CYH2 aac2Δ::URA3/AAC2 fzo1Δ::LEU2/FZO1*	Garipler and Dunn (2013)		
CDD62	*MATα/MATa ura3-1/ura3-1 trp1Δ2/trp1Δ2 ade2-1/ade2-1 leu2-3,112/leu2-3,112 his3-11,15/his3-11,15 can1-100/can1-100 cyh2/CYH2 aac2Δ::URA3/AAC2 fzo1Δ::HIS3/FZO1*	This study	CDD59	replaced *LEU2* at *FZO1* locus with *HIS3* using primers 30/31 and template pRS303
CDD63	*MATα/MATa ura3-1/ura3-1 trp1Δ2/trp1Δ2 ade2-1/ade2-1 leu2-3,112/leu2-3,112 his3-11,15/his3-11,15 can1-100/can1-100 cyh2/CYH2 aac2Δ::URA3/AAC2 fzo1Δ::LEU2/FZO1 pFZO1-CYH2-TRP1*	Garipler and Dunn (2013)		
CDD67	*MATα ura3-1 trp1Δ2 ade2-1 leu2-3,112 his3-11,15 can1-100 cyh2 fzo1Δ::LEU2 aac2Δ::URA3 pFZO1-CYH2-TRP1*	This study	CDD63	sporulation
CDD68	*MATa ura3-1 trp1Δ2 ade2-1 leu2-3,112 his3-11,15 can1-100 aac2Δ::URA3*	Garipler and Dunn (2013)		
CDD71	*MATa ura3-1 trp1Δ2 ade2-1 leu2-3,112 his3-11,15 can1-100 cyh2 fzo1Δ::HIS3 aac2Δ::URA3 pFZO1-CYH2-TRP1*	This study	CDD62	transformed with plasmid b19 (p*FZO1-CYH2-TRP1*), followed by sporulation
CDD72	*MATα ura3-1 trp1Δ2 ade2-1 leu2-3,112 his3-11,15 can1-100 cyh2 fzo1Δ::LEU2 aac2Δ::URA3 dnm1Δ::kanMX4*	This study	CDD67	transformed *dnm1Δ::kanMX4* cassette amplified from CDD29 using primers 50/51, counterselected plasmid b19 (p*FZO1-CYH2-TRP1*) on YEPD containing 10μg/ml CHX

CDD73	*MATα ura3-1 trp1Δ2 ade2-1 leu2-3,112 his3-11,15 can1-100 cyh2 fzo1Δ::LEU2 aac2Δ::URA3 mdv1Δ::kanMX4*	This study	CDD67	transformed *mdv1Δ::kanMX4* cassette amplified from CDD36 using primers 52/53, counterselected plasmid b19 (p*FZO1-CYH2-TRP1*) on YEPD containing 10μg/mL CHX
CDD74	*MATα ura3-1 trp1Δ2 ade2-1 leu2-3,112 his3-11,15 can1-100 cyh2 fzo1Δ::LEU2 aac2Δ::URA3 fis1Δ::kanMX4*	This study	CDD67	transformed *fis1Δ::kanMX4* cassette amplified from CDD38 using primers 54/55, counterselected plasmid b19 (p*FZO1-CYH2-TRP1*) on YEPD containing 10μg/mL CHX
CDD91	*MATa ura3-1 trp1Δ2 ade2-1 leu2-3,112 his3-11,15 can1-100 cyh2 fzo1Δ::HIS3 aac2Δ::URA3 sfa14 (PDR1-14)*	This study	CDD71	plasmid counterselection on YEPD medium containing 10μg/mL CHX
CDD95	*MATa ura3-1 trp1Δ2 ade2-1 leu2-3,112 his3-11,15 can1-100 cyh2 fzo1Δ::HIS3 aac2Δ::URA3 sfa196 (PDR1-196)*	This study	CDD71	irradiated with ultraviolet light at 312nm, followed by plasmid counterselection on YEPD medium containing 10μg/mL CHX
CDD98	*MATa ura3-1 trp1Δ2 ade2-1 leu2-3,112 his3-11,15 can1-100 cyh2 fzo1Δ::HIS3 aac2Δ::URA3 sfa233 (PDR1-233)*	This study	CDD71	irradiated with ultraviolet light at 312nm, followed by plasmid counterselection on YEPD medium containing 10μg/mL CHX
CDD99	*MATa ura3-1 trp1Δ2 ade2-1 leu2-3,112 his3-11,15 can1-100 cyh2 fzo1Δ::HIS3 aac2Δ::URA3 sfa248 (PDR1-248)*	This study	CDD71	irradiated with ultraviolet light at 312nm, followed by plasmid counterselection on YEPD medium containing 10μg/mL CHX
CDD100	*MATa ura3-1 trp1Δ2 ade2-1 leu2-3,112 his3-11,15 can1-100 cyh2 fzo1Δ::HIS3 aac2Δ::URA3 sfa249 (PDR1-249)*	This study	CDD71	irradiated with ultraviolet light at 312nm, followed by plasmid counterselection on YEPD medium containing 10μg/mL CHX
CDD101	*MATa ura3-1 trp1Δ2 ade2-1 leu2-3,112 his3-11,15 can1-100 cyh2 fzo1Δ::HIS3 aac2Δ::URA3 fis1Δ::kanMX4*	This study	CDD71	transformed *fis1Δ::kanMX4* cassette amplified from CDD38 using primers 54/55, counterselected plasmid b19 (p*FZO1-CYH2-TRP1*) on YEPD containing 10μg/mL CHX
CDD104	*MATa ura3-1 trp1Δ2 ade2-1 leu2-3,112 his3-11,15 can1-100 cyh2 fzo1Δ::HIS3 aac2Δ::URA3 sfa142 (PDR1-142)*	This study	CDD71	irradiated with ultraviolet light at 312nm, followed by plasmid counterselection on YEPD medium containing 10μg/mL CHX
CDD105	*MATa ura3-1 trp1Δ2 ade2-1 leu2-3,112 his3-11,15 can1-100 cyh2 fzo1Δ::HIS3 aac2Δ::URA3 sfa159 (PDR3-159)*	This study	CDD71	irradiated with ultraviolet light at 312nm, followed by plasmid counterselection on YEPD medium containing 10μg/mL CHX
CDD116	*MATα/MATa ura3-1/ura3-1 trp1Δ2/trp1Δ2 ade2-1/ade2-1 leu2-3,112/leu2-3,112 his3-11,15/his3-11,15 can1-100/can1-100 cyh2/CYH2 aac2Δ::URA3/AAC2 fzo1Δ::LEU2/FZO1 rvs161Δ::HIS3/RVS161 pFZO1-CYH2-TRP1*	This study	CDD63	deleted *RVS161* with the *HIS3* cassette using primers 66/67 and template pRS303
CDD132	*MATa ura3-1 trp1Δ2 ade2-1 leu2-3,112 his3-11,15 can1-100 cyh2 fzo1Δ::LEU2 pFZO1-CYH2-TRP1*	This study	CDD116	sporulation
CDD342	*MATa ura3-1 trp1Δ2 ade2-1 leu2-3,112 his3-11,15 can1-100 cyh2 fzo1Δ::LEU2 fis1Δ::kanMX4*	This study	CDD132	transformed *fis1Δ::kanMX4* cassette amplified from CDD38 using primers 54/55, lost plasmid b19 (p*FZO1-CYH2-TRP1*)
CDD619	*MATa cry1 lys1 ρ-*	Garipler et al. (2014)		
CDD620	*MATα lys1 ρ-*	This study	RJ98	cells were patched to YEPD + 25μg/ml ethidium bromide for two days, followed by colony purification
CDD638	*MATα/MATa ura3-1/ura3-1 trp1Δ2/trp1Δ2 ade2-1/ade2-1 leu2-3,112/leu2-3,112 his3-11,15/his3-11,15 can1-100/can1-100 pdr1Δ::LEU2/PDR1*	This study	BMA64	deleted *PDR1* with the *LEU2* cassette using primers 208/209 and template pRS305
CDD642	*MATα ura3-1 trp1Δ2 ade2-1 leu2-3,112 his3-11,15 can1-100*	This study	CDD638	sporulation
CDD643	*MATα ura3-1 trp1Δ2 ade2-1 leu2-3,112 his3-11,15 can1-100 pdr1Δ::LEU2*	This study	CDD638	sporulation
CDD646	*MATα ura3-1 trp1Δ2 ade2-1 leu2-3,112 his3-11,15 can1-100 cyh2 pdr1Δ::LEU2*	This study	CDD51	deleted *PDR1* with the *LEU2* cassette using primers 208/209 and template pRS305

CDD658	*MATα ura3-1 trp1Δ2 ade2-1 leu2-3,112 his3-11,15 can1-100 PDR1-249*	This study	CDD643, CDD100	mating, followed by sporulation
CDD664	*MATa ura3-1 trp1Δ2 ade2-1 leu2-3,112 his3-11,15 can1-100 cyh2 fzo1Δ::HIS3 aac2Δ::URA3 PDR1-249 pFZO1-CYH2-TRP1*	This study	CDD646, CDD100	mating, followed by transformation with plasmid b19 (p*FZO1-CYH2-TRP1*) and sporulation
CDD670	*MATα ura3-1 trp1Δ2 ade2-1 leu2-3,112 his3-11,15 can1-100 cyh2 fzo1Δ::HIS3 PDR1-249 pFZO1-CYH2-TRP1*	This study	CDD646, CDD100	mating, followed by transformation with plasmid b19 (p*FZO1-CYH2-TRP1*) and sporulation
CDD672	*MATα ura3-1 trp1Δ2 ade2-1 leu2-3,112 his3-11,15 can1-100 cyh2 fzo1Δ::HIS3 aac2Δ::URA3 pdr1Δ::LEU2 pFZO1-CYH2-TRP1*	This study	CDD646, CDD91	mating, followed by transformation with plasmid b19 (p*FZO1-CYH2-TRP1*) and sporulation
CDD685	*MATa ura3-1 trp1Δ2 ade2-1 leu2-3,112 his3-11,15 can1-100 cyh2 fzo1Δ::HIS3 aac2Δ::URA3 PDR1-249 tom71Δ::LEU2 pFZO1-CYH2-TRP1*	This study	CDD664	deleted *TOM71* with the *LEU2* cassette using primers 488/489 and template pRS305
CDD687	*MATa ura3-1 trp1Δ2 ade2-1 leu2-3,112 his3-11,15 can1-100 cyh2 fzo1Δ::HIS3 aac2Δ::URA3 fis1Δ::kanMX4 pFZO1-CYH2-TRP1*	This study	CDD71	transformed *fis1Δ::kanMX4* cassette amplified from CDD342 using primers 54/55
CDD688	*MATa ura3-1 trp1Δ2 ade2-1 leu2-3,112 his3-11,15 can1-100 cyh2 fzo1Δ::LEU2 fis1Δ::kanMX4 pFZO1-CYH2-TRP1*	This study	CDD132	transformed *fis1Δ::kanMX4* cassette amplified from CDD342 using primers 54/55
CDD692	*MATa ura3-1 trp1Δ2 ade2-1 leu2-3,112 his3-11,15 can1-100 fis1Δ::kanMX4*	This study	CDD642, CDD342	mating, followed by sporulation
CDD696	*MATα ura3-1 trp1Δ2 ade2-1 leu2-3,112 his3-11,15 can1-100 cyh2 pFZO1-CYH2-TRP1*	This study	CDD51	transformed with plasmid b19 (p*FZO1-CYH2-TRP1*)
CDD698	*MATα ura3-1 trp1Δ2 ade2-1 leu2-3,112 his3-11,15 can1-100 cyh2 aac2Δ::URA3 pFZO1-CYH2-TRP1*	This study	CDD58	sporulation, followed by transformation of a CHX^R Ura^+ segregant with plasmid b19 (p*FZO1-CYH2-TRP1*)
CDD703	*MATα/MATa ura3-1/ura3-1 trp1Δ2/trp1Δ2 ade2-1/ade2-1 leu2-3,112/leu2-3,112 his3-11,15/his3-11,15 can1-100/can1-100 cyh2/cyh2 fzo1Δ::HIS3/fzo1Δ::LEU2 aac2Δ::URA3/aac2Δ::URA3 pFZO1-CYH2-TRP1*	This study	CDD67, CDD71	mating
CDD704	*MATα/MATa ura3-1/ura3-1 trp1Δ2/trp1Δ2 ade2-1/ade2-1 leu2-3,112/leu2-3,112 his3-11,15/his3-11,15 can1-100/can1-100 cyh2/cyh2 fzo1Δ::HIS3/fzo1Δ::LEU2 aac2Δ::URA3/aac2Δ::URA3 PDR1-249/PDR1 pFZO1-CYH2-TRP1*	This study	CDD67, CDD664	mating
CDD714	*MATa ura3-1 trp1Δ2 ade2-1 leu2-3,112 his3-11,15 can1-100 cyh2 fzo1Δ::HIS3 aac2Δ::URA3 mdm36Δ::kanMX4 pFZO1-CYH2-TRP1*	This study	CDD71	transformed *mdm36Δ::kanMX4* cassette amplified from CDD42 using primers 86/87
CDD716	*MATα ura3-1 trp1Δ2 ade2-1 leu2-3,112 his3-11,15 can1-100 cyh2 mgm1Δ::kanMX4 aac2Δ::URA3 PDR1-249 pMGM1-CYH2-TRP1*	This study	CDD664, CDD696	mated, lost plasmid b19 on YEPD containing 10μg/mL CHX, deleted *MGM1* with the *kanMX4* cassette using primers 40/41 and template pRS306K, transformed with p*MGM1-CYH2-TRP1* plasmid b86, sporulated, and sequencing of the *PDR1* locus of a resulting haploid
CDD717	*MATα ura3-1 trp1Δ2 ade2-1 leu2-3,112 his3-11,15 can1-100 cyh2 mgm1Δ::kanMX4 aac2Δ::URA3 pMGM1-CYH2-TRP1*	This study	CDD664, CDD696	mated, lost plasmid b19 on YEPD containing 10μg/mL CHX, deleted *MGM1* with the *kanMX4* cassette using primers 40/41 and template pRS306K, transformed with p*MGM1-CYH2-TRP1* plasmid b86, sporulated, and sequencing of the *PDR1* locus of a resulting haploid
CDD744	*MATα/MATa ura3-1/ura3-1 trp1Δ2/trp1Δ2 ade2-1/ade2-1 leu2-3,112/leu2-3,112 his3-11,15/his3-11,15 can1-100/can1-100 aac2Δ::URA3/AAC2 sur4Δ::kanMX4/SUR4*	This study	BMA64-1B, CDD68	mated, deleted *SUR4* with the *kanMX4* cassette using primers 99/100 and template pRS306K
CDD750	*MATa ura3-1 trp1Δ2 ade2-1 leu2-3,112 his3-11,15 can1-100 cyh2 fzo1Δ::HIS3 aac2Δ::URA3 num1Δ::kanMX4 pFZO1-CYH2-TRP1*	This study	CDD71	deleted *NUM1* with the *kanMX4* cassette using primers 585/586 and template pRS306K
CDD768	*MATα/MATa ura3-1/ura3-1 trp1Δ2/trp1Δ2 ade2-1/ade2-1 leu2-3,112/leu2-3,112 his3-11,15/his3-11,15 can1-100/can1-100 cyh2/CYH2 fzo1Δ::HIS3/FZO1 PDR1-249/PDR1 pFZO1-CYH2-TRP1*	This study	CDD670, BMA64-1A	treated CDD670 with 25μg/ml EtBr for two days to force mtDNA loss, tested for failure to proliferate on YEPGE, then mated to BMA64-1A

Baudin-Baillieu, A., E. Guillemet, C. Cullin and F. Lacroute, 1997 Construction of a yeast strain deleted for the TRP1 promoter and coding region that enhances the efficiency of the polymerase chain reaction-disruption method. Yeast 13**:** 353-356.

Garipler, G., and C. D. Dunn, 2013 Defects associated with mitochondrial DNA damage can be mitigated by increased vacuolar pH in Saccharomyces cerevisiae. Genetics 194**:** 285-290.

Garipler, G., N. Mutlu, N. A. Lack and C. D. Dunn, 2014 Deletion of conserved protein phosphatases reverses defects associated with mitochondrial DNA damage in Saccharomyces cerevisiae. Proc Natl Acad Sci U S A 111**:** 1473-1478.

Table S2 - Oligonucleotides used in this study.

Primer	Sequence
1	ATAAGAATGCGGCCGCCCAGGTGACAGAATGTCTGGGTTGAAAG
2	ATAAGAATGCGGCCGCCTTGCTCCTTGTTGTCTTTTAAATGGAG
30	ATGTCTGAAGGAAAACAACAATTCAAAGACAGCAATAAACAGATTGTACTGAGAGTGCAC
31	CTAATCGATGTCTAAATTTATTTCTTCCACCATCAATTTTCTGTGCGGTATTTCACACCG
40	ATGAATGCGAGCCCAGTACGGCTTTTAATTCTGAGAAGACAGATTGTACTGAGAGTGCAC
41	TCATAAATTTTTGGAGACGCCCTTGTAGCTTTTCTTGAAACTGTGCGGTATTTCACACCG
42	GGGCTCGAGGTGTCAGTAAATAACAGAG
43	AATGCGGCCGCCTTAGATGAAGGGTATG
50	GAGAGGAATACGATACAGAGGAAGGC
51	GCTTATTATGACTGATATTCTTTTGATGCATAACG
52	GACTGTTCTGGTACAATTTCCACCTTG
53	GACCTTTACCTTGAGATGCGTCTG
54	CAGTTCAAATAACATGTGTCCATTACCTGTAC
55	AGAAGGCAAAATAGCAGTGCGTTTATATAC
64	GGGACAAACAACGTTGTAAGGAGTTGATCCTAAAGCA
65	GTATTGTTATAAGTCATTGTAATTGTTTGCTCGGC
66	CAAATCCTATTATAAGAAGCCAGAAGAAAGCTGATACAAGAGATTGTACTGAGAGTGCAC
67	ACATAAATGACCGTAAAAAACTAAAGGCAAAAGCATTAATCTGTGCGGTATTTCACACCG
86	CATCAAAACTTGACTTCTTACTCTTTCTCTACG
87	GGATGTGAGTAGTATTTGGACTTCGC
99	ATTCGGCTTTTTTCCGTTTGTTTACGAAACATAAACAGTCAGATTGTACTGAGAGTGCAC
100	TTTTCTTTTTCATTCGCTGTCAAAAATTCTCGCTTCCTATCTGTGCGGTATTTCACACCG
208	CAGCCAAGAATATACAGAAAAGAATCCAAGAAACTGGAAGAGATTGTACTGAGAGTGCAC
209	GGAAGTTTTTGAGAACTTTTATCTATACAAACGTATACGTCTGTGCGGTATTTCACACCG
459	GGCTCTAGATTGATACATATATCCTCAGTTTAGCTTTTTTTACG
460	ATAAGAATGCGGCCGCAGCGATGCTAATCTATGTACACTACGTAAATATC
488	ATCTCTACATACTTGTATATACCGAACATAAGAAGCTCTTAGATTGTACTGAGAGTGCAC
489	TAACTAAAAGTATATATTTGACCAATACCTGACATATCTTCTGTGCGGTATTTCACACCG
535	TGGTTATGCCTTCACCATGA
536	TGAACCATAAACACCATCAGAGA
537	GGTGCCAAGAAGGTTGTCAT
538	AACGGCATCTTCGGTGTAAC
585	AAAGACGCAACGGTCAAGGCTTTCCACGAGACGTTCGAATAGATTGTACTGAGAGTGCAC
586	TATTGTTCTTAATTTACTTAGAGTTATTTAGTTTTTTTAACTGTGCGGTATTTCACACCG

File S1 is available for download at http://www.g3journal.org/lookup/suppl/doi:10.1534/g3.114.010330/-/DC1.
