# Supplementary material for: Activation of the Pleiotropic Drug Resistance Pathway Can Promote Mitochondrial DNA Retention by Fusion-Defective Mitochondria in Saccharomyces cerevisiae
Source: G3 (Bethesda). 2014 May 6;4(7):1247–58. doi: 10.1534/g3.114.010330 (PMC4455774; doi:10.1534/g3.114.010330)
Supplement: Supporting Information [file supp_g3.114.010330_FigureS1.pdf]

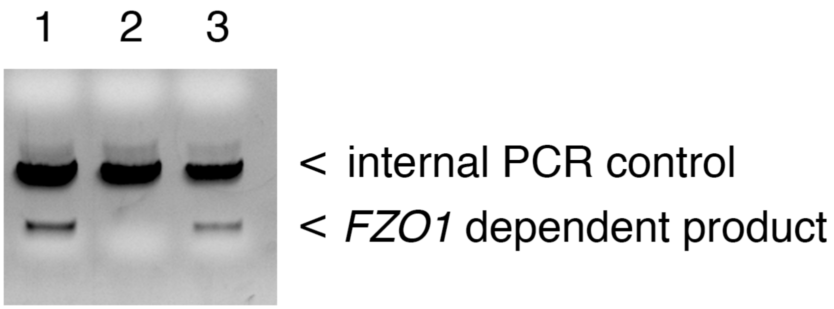

Figure S1 The *PDR1-249* allele permits loss of *FZO1* from cells lacking *AAC2* upon YEPD medium, but not from cells expressing *AAC2*. Genomic DNA was harvested from the following isolates: (1) An example colony from *fzo1Δ cyh2 PDR1-249* strain CDD670, subjected to counter-selection against plasmid b19 (p*FZO1-CYH2*) upon YEPGE containing 3 µg/ml CHX; (2) A colony isolated from *fzo1Δ aac2Δ cyh2 PDR1-249* strain CDD664 lacking plasmid b19 (p*FZO1-CYH2*) following culture on YEPD + 10 µg/ml CHX; and (3) *FZO1* control strain CDD768. A multiplex PCR reaction was performed using primers 54 and 55, amplifying *FIS1* and surrounding sequence (large product), and primers 64 and 65, which amplify a region within the *FZO1* ORF (small product).
