# Supplementary material for: Activation of the Pleiotropic Drug Resistance Pathway Can Promote Mitochondrial DNA Retention by Fusion-Defective Mitochondria in Saccharomyces cerevisiae
Source: G3 (Bethesda). 2014 May 6;4(7):1247–58. doi: 10.1534/g3.114.010330 (PMC4455774; doi:10.1534/g3.114.010330)
Supplement: Supporting Information [file supp_g3.114.010330_FigureS2.pdf]

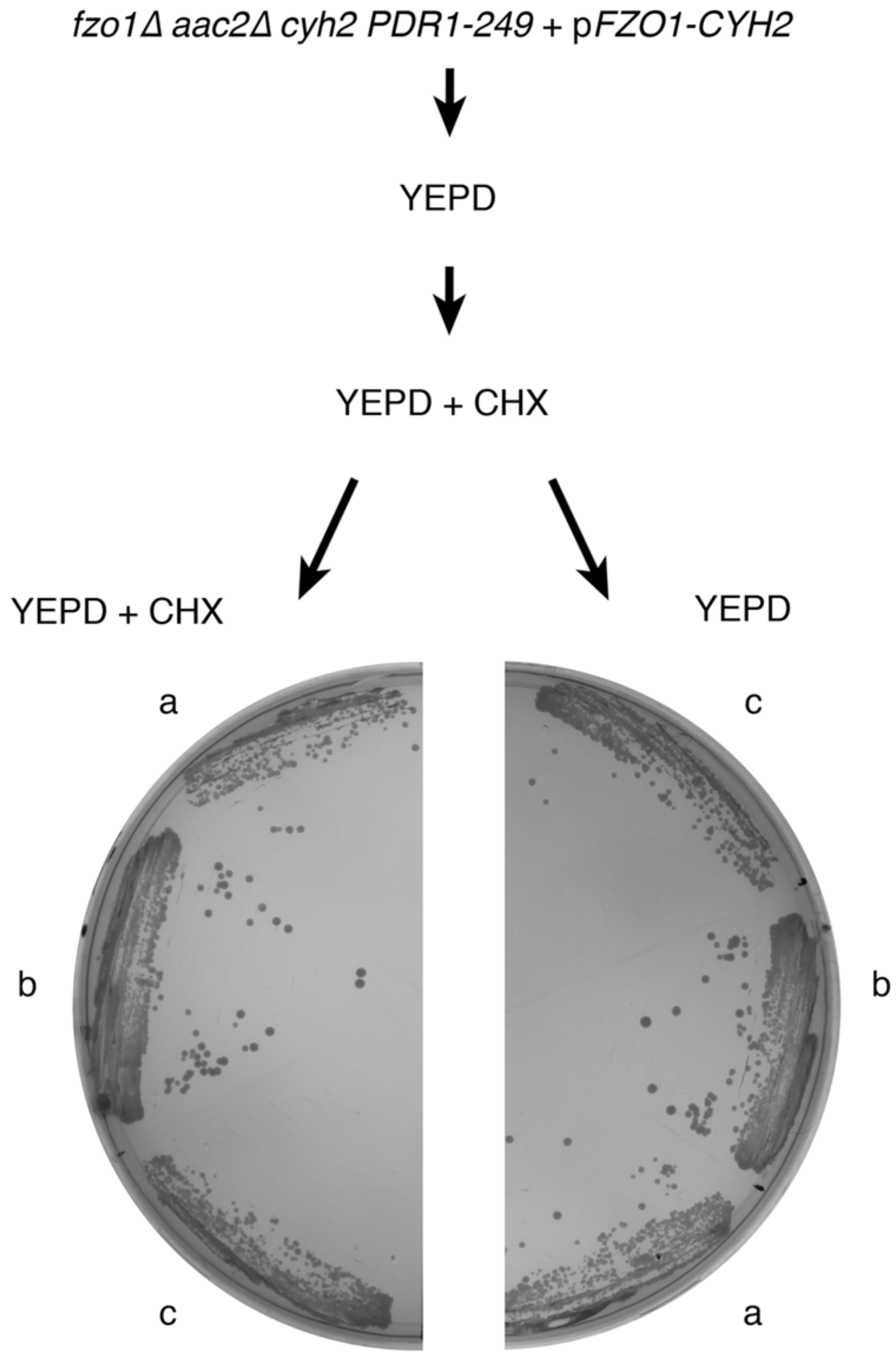


**Figure S2** The presence of cycloheximide is not required for suppression of mtDNA loss from *fzo1Δ aac2Δ* cells by *PDR1-249*. Cells from *fzo1Δ aac2Δ PDR1-249* strain CDD664 carrying plasmid b19 (p*FZO1-CYH2)* were struck to YEPD medium for 1 d, followed by culture on YEPD medium containing 10 µg/ml CHX for 3 d to counter-select against plasmid-borne *FZO1*. Next, cells from three viable *fzo1Δ aac2Δ PDR1-249* colonies (denoted a, b, and c) were struck to both YEPD and to YEPD + CHX, then incubated for a further 3 d.
