# Supplementary material for: Activation of the Pleiotropic Drug Resistance Pathway Can Promote Mitochondrial DNA Retention by Fusion-Defective Mitochondria in Saccharomyces cerevisiae
Source: G3 (Bethesda). 2014 May 6;4(7):1247–58. doi: 10.1534/g3.114.010330 (PMC4455774; doi:10.1534/g3.114.010330)
Supplement: Supporting Information [file supp_g3.114.010330_FigureS3.pdf]

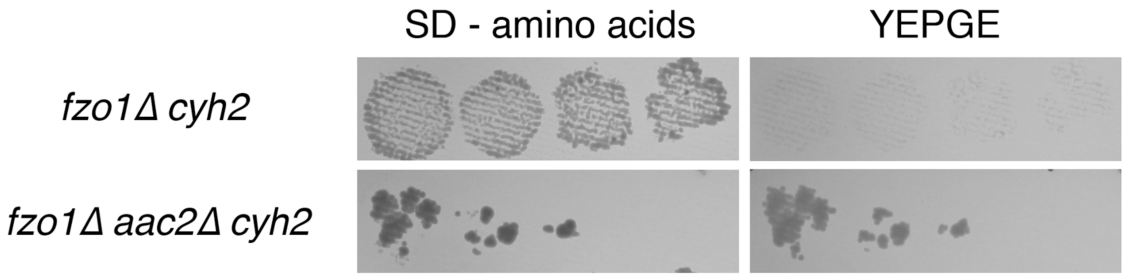

Figure S3 Viable microcolonies of *fzo1Δ aac2Δ* cells contain mtDNA. Microcolonies of strains CDD71 (*fzo1Δ aac2Δ cyh2*) and CDD132 (*fzo1Δ cyh2*) forced to lose plasmid b19 (*pFZO1-CYH2*) in Figure 2A were mated to ρ^- tester strain CDD620. Diploids were replica-plated to SD medium lacking all amino acids in order to demonstrate mating prowess and to YEPGE medium in order to assay the presence of mtDNA. Diploid cells were then incubated for 2 d.
