# Supplementary material for: Activation of the Pleiotropic Drug Resistance Pathway Can Promote Mitochondrial DNA Retention by Fusion-Defective Mitochondria in Saccharomyces cerevisiae
Source: G3 (Bethesda). 2014 May 6;4(7):1247–58. doi: 10.1534/g3.114.010330 (PMC4455774; doi:10.1534/g3.114.010330)
Supplement: Supporting Information [file supp_g3.114.010330_FigureS4.pdf]

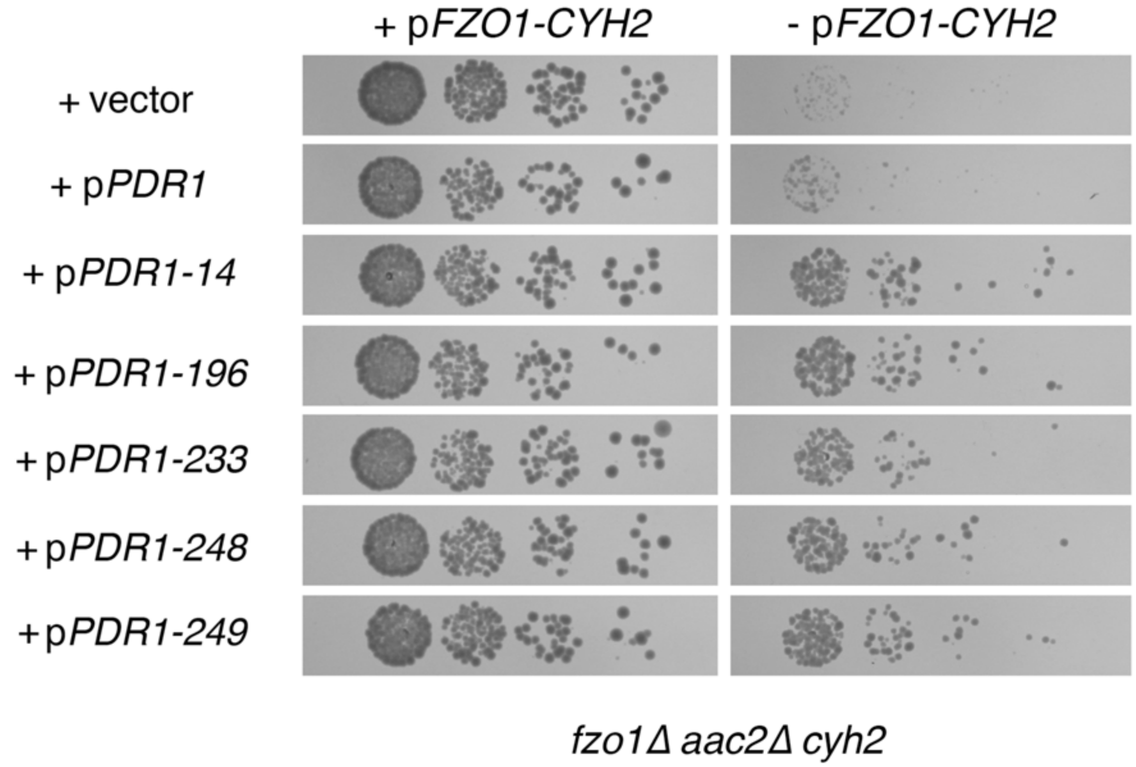

Figure S4 All isolated *PDR1* mutations act as dominant suppressors of the proliferation defect of *fzo1Δ aac2Δ* cells. Strain CDD67 (*fzo1Δ aac2Δ*), also harboring a chromosomal *cyh2* mutation and plasmid b19 (p*FZO1-CYH2*), was transformed with empty vector pRS313, plasmids b60 (p*PDR1*), b61 (p*PDR1-14*), b62 (p*PDR1-196*), b63 (p*PDR1-233*), b64 (p*PDR1-248*), or b65 (p*PDR1-249*). Transformants were treated as in Figure 3C, except that cells not subject to counter-selection using CHX were incubated for 2 d.
