# Supplementary material for: Activation of the Pleiotropic Drug Resistance Pathway Can Promote Mitochondrial DNA Retention by Fusion-Defective Mitochondria in Saccharomyces cerevisiae
Source: G3 (Bethesda). 2014 May 6;4(7):1247–58. doi: 10.1534/g3.114.010330 (PMC4455774; doi:10.1534/g3.114.010330)
Supplement: Supporting Information [file supp_g3.114.010330_FigureS5.pdf]

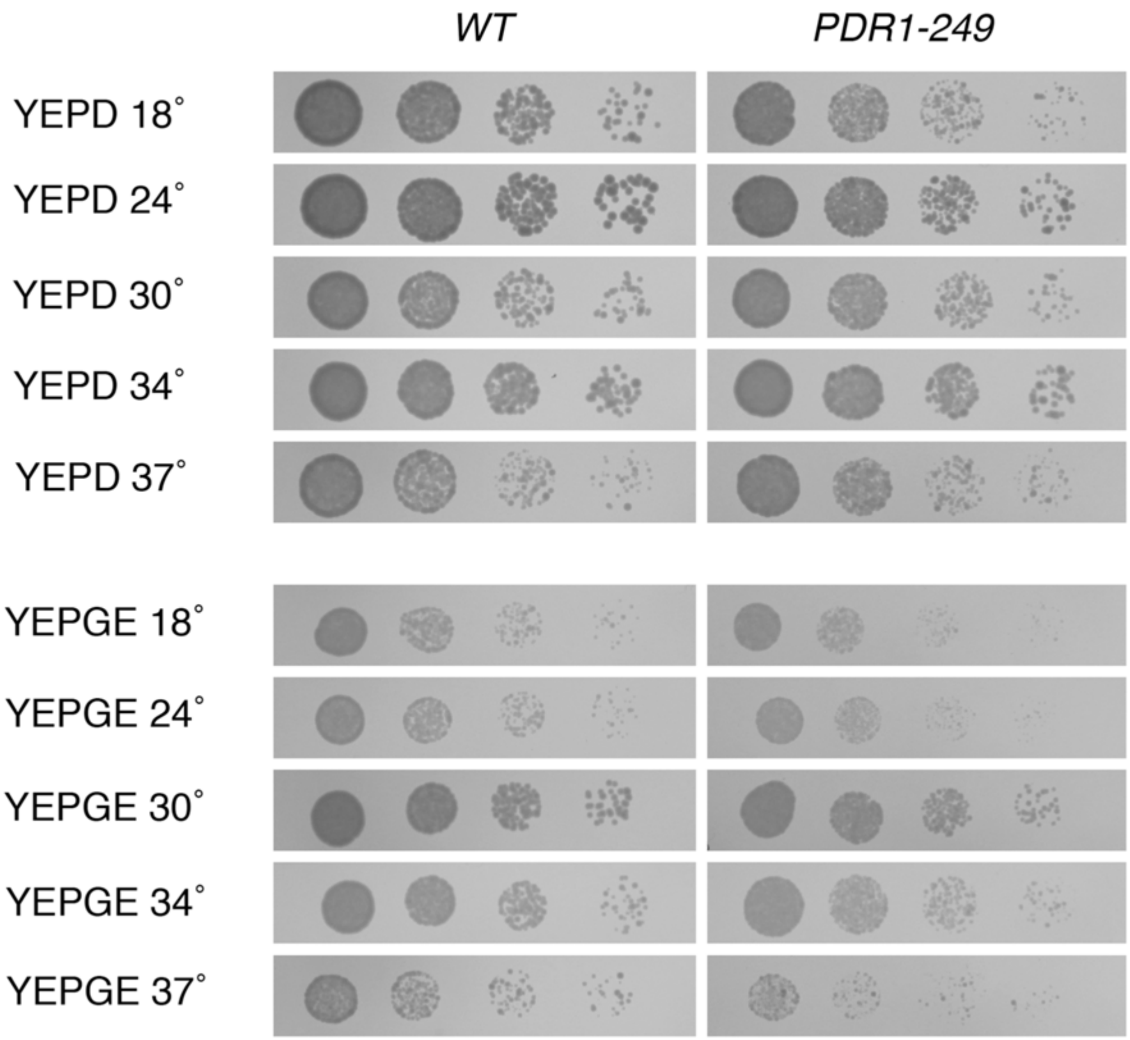

Figure S5 *PDR1-249* cells exhibit minimal proliferation defects on fermentable or non-fermentable medium. Strains CDD642 (*WT*) and CDD658 (*PDR1-249*) were cultured in YEPD overnight at 30˚, then serially diluted and plated at the indicated temperature on either YEPD or YEPGE medium. Cells were incubated for 1 d on YEPD at 30˚, 34˚, and 37˚. Strains were incubated for 2 d on YEPD at 24˚ and on YEPGE at 24˚, 30˚, and 34˚. Cultures were incubated for 3 d on YEPD at 18˚ and on YEPGE at 18˚ and 37˚.
