# Supplementary material for: Activation of the Pleiotropic Drug Resistance Pathway Can Promote Mitochondrial DNA Retention by Fusion-Defective Mitochondria in Saccharomyces cerevisiae
Source: G3 (Bethesda). 2014 May 6;4(7):1247–58. doi: 10.1534/g3.114.010330 (PMC4455774; doi:10.1534/g3.114.010330)
Supplement: Supporting Information [file supp_g3.114.010330_FigureS6.pdf]

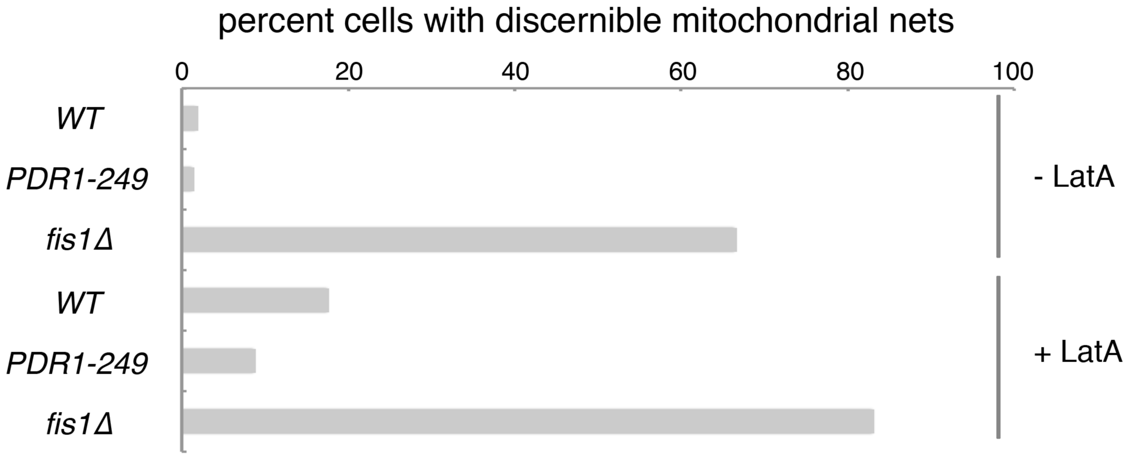

Figure S6 Quantification of mitochondrial networks following latrunculin A treatment. The number of cells containing mitochondrial networks from strains CDD642 (*WT*), CDD658 (*PDR1-249*), and CDD692 (*fis1Δ*), stained with MitoTracker Green FM and shown in Figure 4, was quantified (n>200 cells).
