# Supplementary material for: Activation of the Pleiotropic Drug Resistance Pathway Can Promote Mitochondrial DNA Retention by Fusion-Defective Mitochondria in Saccharomyces cerevisiae
Source: G3 (Bethesda). 2014 May 6;4(7):1247–58. doi: 10.1534/g3.114.010330 (PMC4455774; doi:10.1534/g3.114.010330)
Supplement: Supporting Information [file supp_g3.114.010330_FigureS7.pdf]

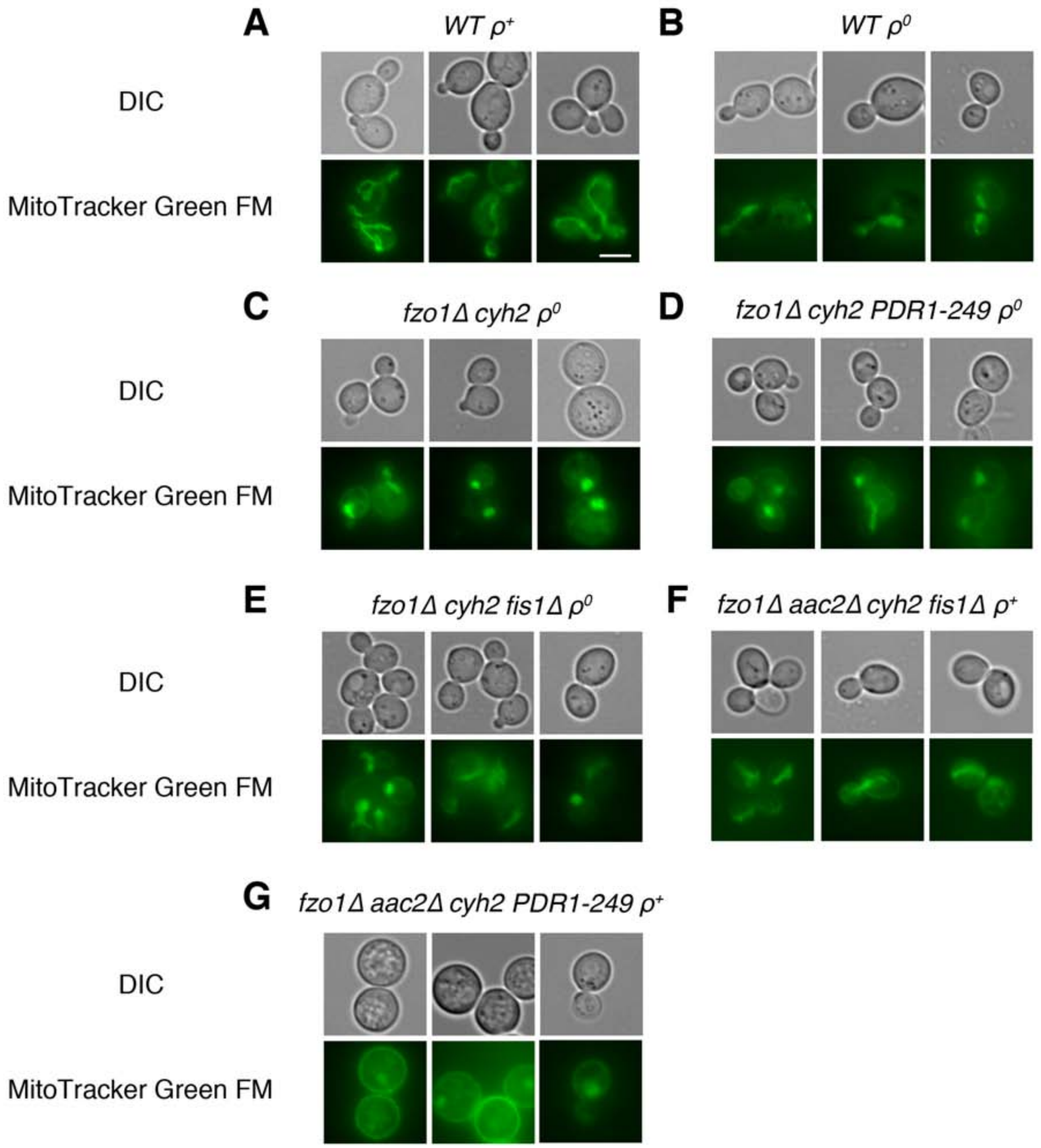

Figure S7 Examination of the morphology of fusion-defective mitochondria. The following strains cultured in YEPD within the logarithmic phase of growth were stained with MitoTracker Green FM and visualized by fluorescence microscopy: (A) CDD642 containing mtDNA (*WT* ρ^+). (B) CDD642 lacking mtDNA following EtBr treatment (*WT* ρ^0). (C) CDD132 lacking mtDNA following counter-selection for p*FZO1-CYH2* plasmid b19 (*fzo1Δ cyh2* ρ^0). (D) CDD670 lacking mtDNA following counter-selection for plasmid b19 (*fzo1Δ cyh2 PDR1-249* ρ^0). (E) CDD688 lacking mtDNA following EtBr treatment and lacking plasmid b19 following counter-selection (*fzo1Δ cyh2Δ fis1Δ* ρ^0). (F) CDD687 lacking plasmid b19 following counter-selection (*fzo1Δ aac2Δ cyh2 fis1Δ* ρ^+). (G) CDD664 lacking plasmid b19 following counter-selection (*fzo1Δ aac2Δ cyh2 fis1Δ* ρ^+). Bar, 5 µm.
