# Supplementary material for: Activation of the Pleiotropic Drug Resistance Pathway Can Promote Mitochondrial DNA Retention by Fusion-Defective Mitochondria in Saccharomyces cerevisiae
Source: G3 (Bethesda). 2014 May 6;4(7):1247–58. doi: 10.1534/g3.114.010330 (PMC4455774; doi:10.1534/g3.114.010330)
Supplement: Supporting Information [file supp_g3.114.010330_FigureS8.pdf]

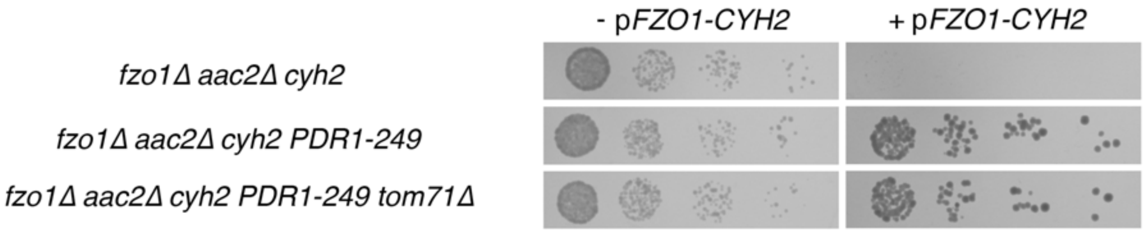

Figure S8 *TOM71* is not required for suppression of mtDNA loss from *fzo1Δ aac2Δ* cells by PDR pathway activation. Strains CDD71 (*fzo1Δ aac2Δ*), CDD664 (*fzo1Δ aac2Δ PDR1-249*), and CDD685 (*fzo1Δ aac2Δ PDR1-249 tom71Δ*), each containing a *cyh2* mutation and plasmid b19 (p*FZO1-CYH2*) were treated as in Figure 2A.
