# Supplementary material for: Activation of the Pleiotropic Drug Resistance Pathway Can Promote Mitochondrial DNA Retention by Fusion-Defective Mitochondria in Saccharomyces cerevisiae
Source: G3 (Bethesda). 2014 May 6;4(7):1247–58. doi: 10.1534/g3.114.010330 (PMC4455774; doi:10.1534/g3.114.010330)
Supplement: Supporting Information [file supp_g3.114.010330_FigureS9.pdf]

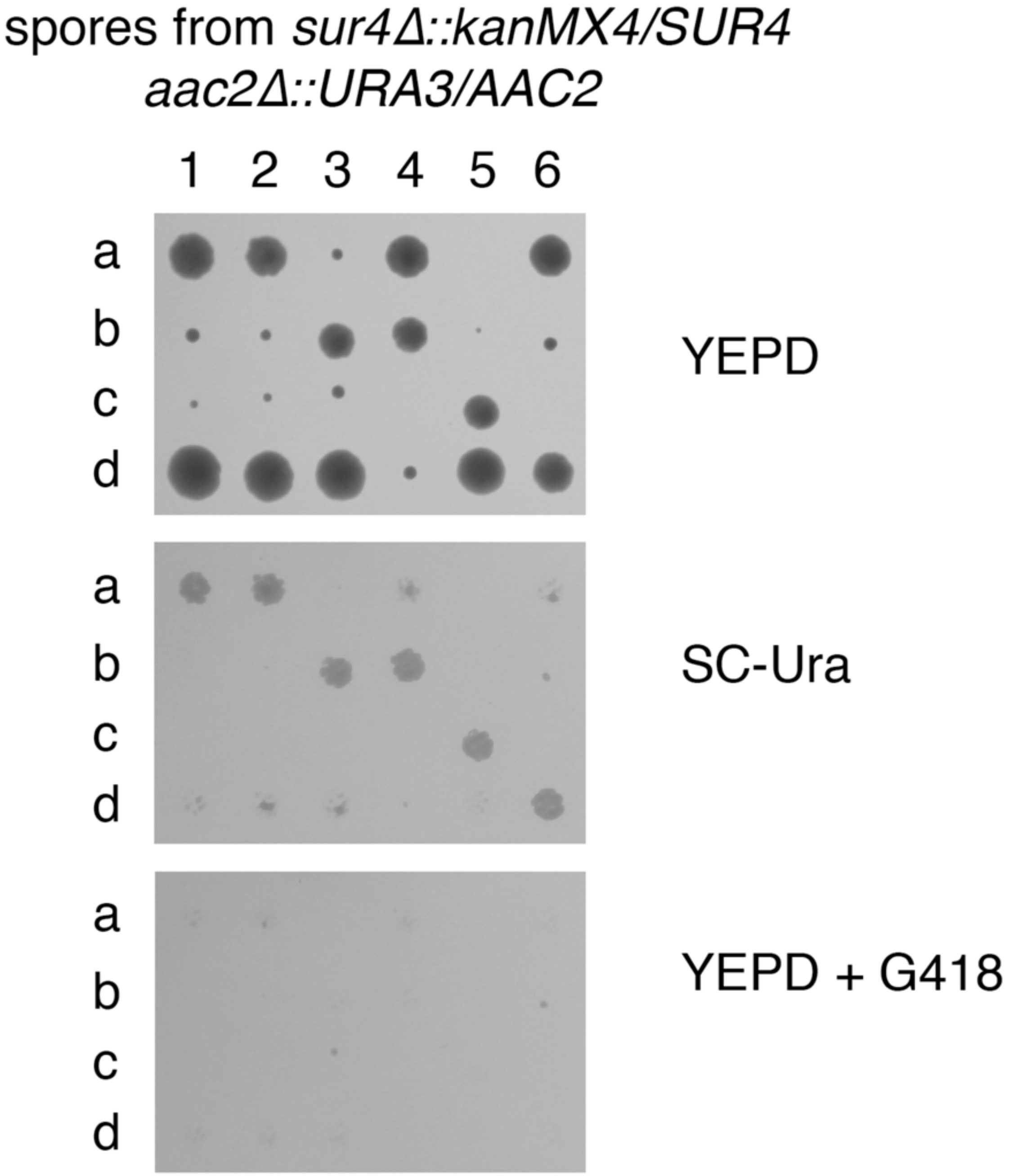

Figure S9 Mutants lacking *sur4Δ* derived from the W303 background of *S. cerevisiae* exhibit a significant proliferation defect. Strain CDD744 (*sur4Δ::kanMX4/SUR4 aac2Δ::URA3/AAC2*) was sporulated, then tetrads were dissected onto YEPD medium and incubated for 4 d. Genotyping was performed by replica plating to SC medium lacking uracil (SC-Ura) or to YEPD containing 200 µg/ml G418, followed by incubation for 1 d.
