# Supplementary material for: Activation of the Pleiotropic Drug Resistance Pathway Can Promote Mitochondrial DNA Retention by Fusion-Defective Mitochondria in Saccharomyces cerevisiae
Source: G3 (Bethesda). 2014 May 6;4(7):1247–58. doi: 10.1534/g3.114.010330 (PMC4455774; doi:10.1534/g3.114.010330)
Supplement: Supporting Information [file supp_g3.114.010330_TableS1.pdf]

Table S1 - Strain genotypes and construction.

Strain	Genotype	Source	Parental strain(s)	Method Used
BMA64-1A	*MATa ura3-1 trp1Δ2 ade2-1 leu2-3,112 his3-11,15 can1-100*	Baudin-Baillieu et al. (1997)		
BMA64-1B	*MATα ura3-1 trp1Δ2 ade2-1 leu2-3,112 his3-11,15 can1-100*	Baudin-Baillieu et al. (1997)		
BMA64	*MATα/MATa ura3-1/ura3-1 trp1Δ2/trp1Δ2 ade2-1/ade2-1 leu2-3,112/leu2-3,112 his3-11,15/his3-11,15 can1-100/can1-100*	Baudin-Baillieu et al. (1997)		
RJ98	*MATα lys1*	Robert Jensen		
CDD29	*MATa leu2Δ0 met15Δ0 ura3Δ0 his3Δ1 dnm1Δ::kanMX4*	EUROSCARF Y01489		
CDD36	*MATa leu2Δ0 met15Δ0 ura3Δ0 his3Δ1 mdv1Δ::kanMX4*	EUROSCARF Y01311		
CDD38	*MATa leu2Δ0 met15Δ0 ura3Δ0 his3Δ1 fis1Δ::kanMX4*	EUROSCARF Y01458		
CDD42	*MATa leu2Δ0 met15Δ0 ura3Δ0 his3Δ1 mdm36Δ::kanMX4*	EUROSCARF Y07401		
CDD51	*MATα ura3-1 trp1Δ2 ade2-1 leu2-3,112 his3-11,15 can1-100 cyh2*	Garipler and Dunn (2013)		
CDD53	*MATα/MATa ura3-1/ura3-1 trp1Δ2/trp1Δ2 ade2-1/ade2-1 leu2-3,112/leu2-3,112 his3-11,15/his3-11,15 can1-100/can1-100 cyh2/CYH2*	Garipler and Dunn (2013)		
CDD58	*MATα/MATa ura3-1/ura3-1 trp1Δ2/trp1Δ2 ade2-1/ade2-1 leu2-3,112/leu2-3,112 his3-11,15/his3-11,15 can1-100/can1-100 cyh2/CYH2 aac2Δ::URA3/AAC2*	Garipler and Dunn (2013)		
CDD59	*MATα/MATa ura3-1/ura3-1 trp1Δ2/trp1Δ2 ade2-1/ade2-1 leu2-3,112/leu2-3,112 his3-11,15/his3-11,15 can1-100/can1-100 cyh2/CYH2 aac2Δ::URA3/AAC2 fzo1Δ::LEU2/FZO1*	Garipler and Dunn (2013)		
CDD62	*MATα/MATa ura3-1/ura3-1 trp1Δ2/trp1Δ2 ade2-1/ade2-1 leu2-3,112/leu2-3,112 his3-11,15/his3-11,15 can1-100/can1-100 cyh2/CYH2 aac2Δ::URA3/AAC2 fzo1Δ::HIS3/FZO1*	This study	CDD59	replaced *LEU2* at *FZO1* locus with *HIS3* using primers 30/31 and template pRS303
CDD63	*MATα/MATa ura3-1/ura3-1 trp1Δ2/trp1Δ2 ade2-1/ade2-1 leu2-3,112/leu2-3,112 his3-11,15/his3-11,15 can1-100/can1-100 cyh2/CYH2 aac2Δ::URA3/AAC2 fzo1Δ::LEU2/FZO1 pFZO1-CYH2-TRP1*	Garipler and Dunn (2013)		
CDD67	*MATα ura3-1 trp1Δ2 ade2-1 leu2-3,112 his3-11,15 can1-100 cyh2 fzo1Δ::LEU2 aac2Δ::URA3 pFZO1-CYH2-TRP1*	This study	CDD63	sporulation
CDD68	*MATa ura3-1 trp1Δ2 ade2-1 leu2-3,112 his3-11,15 can1-100 aac2Δ::URA3*	Garipler and Dunn (2013)		
CDD71	*MATa ura3-1 trp1Δ2 ade2-1 leu2-3,112 his3-11,15 can1-100 cyh2 fzo1Δ::HIS3 aac2Δ::URA3 pFZO1-CYH2-TRP1*	This study	CDD62	transformed with plasmid b19 (p*FZO1-CYH2-TRP1*), followed by sporulation
CDD72	*MATα ura3-1 trp1Δ2 ade2-1 leu2-3,112 his3-11,15 can1-100 cyh2 fzo1Δ::LEU2 aac2Δ::URA3 dnm1Δ::kanMX4*	This study	CDD67	transformed *dnm1Δ::kanMX4* cassette amplified from CDD29 using primers 50/51, counterselected plasmid b19 (p*FZO1-CYH2-TRP1*) on YEPD containing 10μg/ml CHX

CDD73	*MATα ura3-1 trp1Δ2 ade2-1 leu2-3,112 his3-11,15 can1-100 cyh2 fzo1Δ::LEU2 aac2Δ::URA3 mdv1Δ::kanMX4*	This study	CDD67	transformed *mdv1Δ::kanMX4* cassette amplified from CDD36 using primers 52/53, counterselected plasmid b19 (p*FZO1-CYH2-TRP1*) on YEPD containing 10µg/mL CHX
CDD74	*MATα ura3-1 trp1Δ2 ade2-1 leu2-3,112 his3-11,15 can1-100 cyh2 fzo1Δ::LEU2 aac2Δ::URA3 fis1Δ::kanMX4*	This study	CDD67	transformed *fis1Δ::kanMX4* cassette amplified from CDD38 using primers 54/55, counterselected plasmid b19 (p*FZO1-CYH2-TRP1*) on YEPD containing 10µg/mL CHX
CDD91	*MATa ura3-1 trp1Δ2 ade2-1 leu2-3,112 his3-11,15 can1-100 cyh2 fzo1Δ::HIS3 aac2Δ::URA3 sfa14 (PDR1-14)*	This study	CDD71	plasmid counterselection on YEPD medium containing 10µg/mL CHX
CDD95	*MATa ura3-1 trp1Δ2 ade2-1 leu2-3,112 his3-11,15 can1-100 cyh2 fzo1Δ::HIS3 aac2Δ::URA3 sfa196 (PDR1-196)*	This study	CDD71	irradiated with ultraviolet light at 312nm, followed by plasmid counterselection on YEPD medium containing 10µg/mL CHX
CDD98	*MATa ura3-1 trp1Δ2 ade2-1 leu2-3,112 his3-11,15 can1-100 cyh2 fzo1Δ::HIS3 aac2Δ::URA3 sfa233 (PDR1-233)*	This study	CDD71	irradiated with ultraviolet light at 312nm, followed by plasmid counterselection on YEPD medium containing 10µg/mL CHX
CDD99	*MATa ura3-1 trp1Δ2 ade2-1 leu2-3,112 his3-11,15 can1-100 cyh2 fzo1Δ::HIS3 aac2Δ::URA3 sfa248 (PDR1-248)*	This study	CDD71	irradiated with ultraviolet light at 312nm, followed by plasmid counterselection on YEPD medium containing 10µg/mL CHX
CDD100	*MATa ura3-1 trp1Δ2 ade2-1 leu2-3,112 his3-11,15 can1-100 cyh2 fzo1Δ::HIS3 aac2Δ::URA3 sfa249 (PDR1-249)*	This study	CDD71	irradiated with ultraviolet light at 312nm, followed by plasmid counterselection on YEPD medium containing 10µg/mL CHX
CDD101	*MATa ura3-1 trp1Δ2 ade2-1 leu2-3,112 his3-11,15 can1-100 cyh2 fzo1Δ::HIS3 aac2Δ::URA3 fis1Δ::kanMX4*	This study	CDD71	transformed *fis1Δ::kanMX4* cassette amplified from CDD38 using primers 54/55, counterselected plasmid b19 (p*FZO1-CYH2-TRP1*) on YEPD containing 10µg/mL CHX
CDD104	*MATa ura3-1 trp1Δ2 ade2-1 leu2-3,112 his3-11,15 can1-100 cyh2 fzo1Δ::HIS3 aac2Δ::URA3 sfa142 (PDR1-142)*	This study	CDD71	irradiated with ultraviolet light at 312nm, followed by plasmid counterselection on YEPD medium containing 10µg/mL CHX
CDD105	*MATa ura3-1 trp1Δ2 ade2-1 leu2-3,112 his3-11,15 can1-100 cyh2 fzo1Δ::HIS3 aac2Δ::URA3 sfa159 (PDR3-159)*	This study	CDD71	irradiated with ultraviolet light at 312nm, followed by plasmid counterselection on YEPD medium containing 10µg/mL CHX
CDD116	*MATα/MATa ura3-1/ura3-1 trp1Δ2/trp1Δ2 ade2-1/ade2-1 leu2-3,112/leu2-3,112 his3-11,15/his3-11,15 can1-100/can1-100 cyh2/CYH2 aac2Δ::URA3/AAC2 fzo1Δ::LEU2/FZO1 rvs161Δ::HIS3/RVS161 pFZO1-CYH2-TRP1*	This study	CDD63	deleted *RVS161* with the *HIS3* cassette using primers 66/67 and template pRS303
CDD132	*MATa ura3-1 trp1Δ2 ade2-1 leu2-3,112 his3-11,15 can1-100 cyh2 fzo1Δ::LEU2 pFZO1-CYH2-TRP1*	This study	CDD116	sporulation
CDD342	*MATa ura3-1 trp1Δ2 ade2-1 leu2-3,112 his3-11,15 can1-100 cyh2 fzo1Δ::LEU2 fis1Δ::kanMX4*	This study	CDD132	transformed *fis1Δ::kanMX4* cassette amplified from CDD38 using primers 54/55, lost plasmid b19 (p*FZO1-CYH2-TRP1*)
CDD619	*MATa cry1 lys1 ρ-*	Garipler et al. (2014)		
CDD620	*MATα lys1 ρ-*	This study	RJ98	cells were patched to YEPD + 25µg/ml ethidium bromide for two days, followed by colony purification
CDD638	*MATα/MATa ura3-1/ura3-1 trp1Δ2/trp1Δ2 ade2-1/ade2-1 leu2-3,112/leu2-3,112 his3-11,15/his3-11,15 can1-100/can1-100 pdr1Δ::LEU2/PDR1*	This study	BMA64	deleted *PDR1* with the *LEU2* cassette using primers 208/209 and template pRS305
CDD642	*MATα ura3-1 trp1Δ2 ade2-1 leu2-3,112 his3-11,15 can1-100*	This study	CDD638	sporulation
CDD643	*MATα ura3-1 trp1Δ2 ade2-1 leu2-3,112 his3-11,15 can1-100 pdr1Δ::LEU2*	This study	CDD638	sporulation
CDD646	*MATα ura3-1 trp1Δ2 ade2-1 leu2-3,112 his3-11,15 can1-100 cyh2 pdr1Δ::LEU2*	This study	CDD51	deleted *PDR1* with the *LEU2* cassette using primers 208/209 and template pRS305

CDD658	MATα ura3-1 trp1Δ2 ade2-1 leu2-3,112 his3-11,15 can1-100 PDR1-249	This study	CDD643, CDD100	mating, followed by sporulation
CDD664	MATa ura3-1 trp1Δ2 ade2-1 leu2-3,112 his3-11,15 can1-100 cyh2 fzo1Δ::HIS3 aac2Δ::URA3 PDR1-249 pFZO1-CYH2-TRP1	This study	CDD646, CDD100	mating, followed by transformation with plasmid b19 (pFZO1-CYH2-TRP1) and sporulation
CDD670	MATα ura3-1 trp1Δ2 ade2-1 leu2-3,112 his3-11,15 can1-100 cyh2 fzo1Δ::HIS3 PDR1-249 pFZO1-CYH2-TRP1	This study	CDD646, CDD100	mating, followed by transformation with plasmid b19 (pFZO1-CYH2-TRP1) and sporulation
CDD672	MATα ura3-1 trp1Δ2 ade2-1 leu2-3,112 his3-11,15 can1-100 cyh2 fzo1Δ::HIS3 aac2Δ::URA3 pdr1Δ::LEU2 pFZO1-CYH2-TRP1	This study	CDD646, CDD91	mating, followed by transformation with plasmid b19 (pFZO1-CYH2-TRP1) and sporulation
CDD685	MATa ura3-1 trp1Δ2 ade2-1 leu2-3,112 his3-11,15 can1-100 cyh2 fzo1Δ::HIS3 aac2Δ::URA3 PDR1-249 tom71Δ::LEU2 pFZO1-CYH2-TRP1	This study	CDD664	deleted TOM71 with the LEU2 cassette using primers 488/489 and template pRS305
CDD687	MATa ura3-1 trp1Δ2 ade2-1 leu2-3,112 his3-11,15 can1-100 cyh2 fzo1Δ::HIS3 aac2Δ::URA3 fis1Δ::kanMX4 pFZO1-CYH2-TRP1	This study	CDD71	transformed fis1Δ::kanMX4 cassette amplified from CDD342 using primers 54/55
CDD688	MATa ura3-1 trp1Δ2 ade2-1 leu2-3,112 his3-11,15 can1-100 cyh2 fzo1Δ::LEU2 fis1Δ::kanMX4 pFZO1-CYH2-TRP1	This study	CDD132	transformed fis1Δ::kanMX4 cassette amplified from CDD342 using primers 54/55
CDD692	MATa ura3-1 trp1Δ2 ade2-1 leu2-3,112 his3-11,15 can1-100 fis1Δ::kanMX4	This study	CDD642, CDD342	mating, followed by sporulation
CDD696	MATα ura3-1 trp1Δ2 ade2-1 leu2-3,112 his3-11,15 can1-100 cyh2 pFZO1-CYH2-TRP1	This study	CDD51	transformed with plasmid b19 (pFZO1-CYH2-TRP1)
CDD698	MATα ura3-1 trp1Δ2 ade2-1 leu2-3,112 his3-11,15 can1-100 cyh2 aac2Δ::URA3 pFZO1-CYH2-TRP1	This study	CDD58	sporulation, followed by transformation of a CHX^R Ura^+ segregant with plasmid b19 (pFZO1-CYH2-TRP1)
CDD703	MATα/MATa ura3-1/ura3-1 trp1Δ2/trp1Δ2 ade2-1/ade2-1 leu2-3,112/leu2-3,112 his3-11,15/his3-11,15 can1-100/can1-100 cyh2/cyh2 fzo1Δ::HIS3/fzo1Δ::LEU2 aac2Δ::URA3/aac2Δ::URA3 pFZO1-CYH2-TRP1	This study	CDD67, CDD71	mating
CDD704	MATα/MATa ura3-1/ura3-1 trp1Δ2/trp1Δ2 ade2-1/ade2-1 leu2-3,112/leu2-3,112 his3-11,15/his3-11,15 can1-100/can1-100 cyh2/cyh2 fzo1Δ::HIS3/fzo1Δ::LEU2 aac2Δ::URA3/aac2Δ::URA3 PDR1-249/PDR1 pFZO1-CYH2-TRP1	This study	CDD67, CDD664	mating
CDD714	MATa ura3-1 trp1Δ2 ade2-1 leu2-3,112 his3-11,15 can1-100 cyh2 fzo1Δ::HIS3 aac2Δ::URA3 mdm36Δ::kanMX4 pFZO1-CYH2-TRP1	This study	CDD71	transformed mdm36Δ::kanMX4 cassette amplified from CDD42 using primers 86/87
CDD716	MATα ura3-1 trp1Δ2 ade2-1 leu2-3,112 his3-11,15 can1-100 cyh2 mgm1Δ::kanMX4 aac2Δ::URA3 PDR1-249 pMGM1-CYH2-TRP1	This study	CDD664, CDD696	mated, lost plasmid b19 on YEPD containing 10μg/mL CHX, deleted MGM1 with the kanMX4 cassette using primers 40/41 and template pRS306K, transformed with pMGM1-CYH2-TRP1 plasmid b86, sporulated, and sequencing of the PDR1 locus of a resulting haploid
CDD717	MATα ura3-1 trp1Δ2 ade2-1 leu2-3,112 his3-11,15 can1-100 cyh2 mgm1Δ::kanMX4 aac2Δ::URA3 pMGM1-CYH2-TRP1	This study	CDD664, CDD696	mated, lost plasmid b19 on YEPD containing 10μg/mL CHX, deleted MGM1 with the kanMX4 cassette using primers 40/41 and template pRS306K, transformed with pMGM1-CYH2-TRP1 plasmid b86, sporulated, and sequencing of the PDR1 locus of a resulting haploid
CDD744	MATα/MATa ura3-1/ura3-1 trp1Δ2/trp1Δ2 ade2-1/ade2-1 leu2-3,112/leu2-3,112 his3-11,15/his3-11,15 can1-100/can1-100 aac2Δ::URA3/AAC2 sur4Δ::kanMX4/SUR4	This study	BMA64-1B, CDD68	mated, deleted SUR4 with the kanMX4 cassette using primers 99/100 and template pRS306K
CDD750	MATa ura3-1 trp1Δ2 ade2-1 leu2-3,112 his3-11,15 can1-100 cyh2 fzo1Δ::HIS3 aac2Δ::URA3 num1Δ::kanMX4 pFZO1-CYH2-TRP1	This study	CDD71	deleted NUM1 with the kanMX4 cassette using primers 585/586 and template pRS306K
CDD768	MATα/MATa ura3-1/ura3-1 trp1Δ2/trp1Δ2 ade2-1/ade2-1 leu2-3,112/leu2-3,112 his3-11,15/his3-11,15 can1-100/can1-100 cyh2/CYH2 fzo1Δ::HIS3/FZO1 PDR1-249/PDR1 pFZO1-CYH2-TRP1	This study	CDD670, BMA64-1A	treated CDD670 with 25μg/ml EtBr for two days to force mtDNA loss, tested for failure to proliferate on YEPGE, then mated to BMA64-1A

Baudin-Baillieu, A., E. Guillemet, C. Cullin and F. Lacroute, 1997 Construction of a yeast strain deleted for the TRP1 promoter and coding region that enhances the efficiency of the polymerase chain reaction-disruption method. Yeast 13**:** 353-356.

Garipler, G., and C. D. Dunn, 2013 Defects associated with mitochondrial DNA damage can be mitigated by increased vacuolar pH in Saccharomyces cerevisiae. Genetics 194**:** 285-290.

Garipler, G., N. Mutlu, N. A. Lack and C. D. Dunn, 2014 Deletion of conserved protein phosphatases reverses defects associated with mitochondrial DNA damage in Saccharomyces cerevisiae. Proc Natl Acad Sci U S A 111**:** 1473-1478.
