# Supplementary material for: Activation of the Pleiotropic Drug Resistance Pathway Can Promote Mitochondrial DNA Retention by Fusion-Defective Mitochondria in Saccharomyces cerevisiae
Source: G3 (Bethesda). 2014 May 6;4(7):1247–58. doi: 10.1534/g3.114.010330 (PMC4455774; doi:10.1534/g3.114.010330)
Supplement: Supporting Information [file supp_g3.114.010330_TableS2.pdf]

Table S2 - Oligonucleotides used in this study.

Primer	Sequence
1	ATAAGAATGCGGCCGCCCAGGTGACAGAATGTCTGGGTTGAAAG
2	ATAAGAATGCGGCCGCCTTGCTCCTTGTTGTCTTTTAAATGGAG
30	ATGTCTGAAGGAAAACAACAATTCAAAGACAGCAATAAACAGATTGTACTGAGAGTGCAC
31	CTAATCGATGTCTAAATTTATTTCTTCCACCATCAATTTTCTGTGCGGTATTTCACACCG
40	ATGAATGCGAGCCCAGTACGGCTTTTAATTCTGAGAAGACAGATTGTACTGAGAGTGCAC
41	TCATAAATTTTTGGAGACGCCCTTGTAGCTTTTCTTGAAACTGTGCGGTATTTCACACCG
42	GGGCTCGAGGTGTCAGTAAATAACAGAG
43	AATGCGGCCGCCTTAGATGAAGGGTATG
50	GAGAGGAATACGATACAGAGGAAGGC
51	GCTTATTATGACTGATATTCTTTTGATGCATAACG
52	GACTGTTCTGGTACAATTTCCACCTTG
53	GACCTTTACCTTGAGATGCGTCTG
54	CAGTTCAAATAACATGTGTCCATTACCTGTAC
55	AGAAGGCAAAATAGCAGTGCGTTTATATAC
64	GGGACAAACAACGTTGTAAGGAGTTGATCCTAAAGCA
65	GTATTGTTATAAGTCATTGTAATTGTTTGCTCGGC
66	CAAATCCTATTATAAGAAGCCAGAAGAAAGCTGATACAAGAGATTGTACTGAGAGTGCAC
67	ACATAAATGACCGTAAAAAACTAAAGGCAAAAGCATTAATCTGTGCGGTATTTCACACCG
86	CATCAAAACTTGACTTCTTACTCTTTCTCTACG
87	GGATGTGAGTAGTATTTGGACTTCGC
99	ATTCGGCTTTTTTCCGTTTGTTTACGAAACATAAACAGTCAGATTGTACTGAGAGTGCAC
100	TTTTCTTTTTCATTCGCTGTCAAAAATTCTCGCTTCCTATCTGTGCGGTATTTCACACCG
208	CAGCCAAGAATATACAGAAAAGAATCCAAGAAACTGGAAGAGATTGTACTGAGAGTGCAC
209	GGAAGTTTTTGAGAACTTTTATCTATACAAACGTATACGTCTGTGCGGTATTTCACACCG
459	GGCTCTAGATTGATACATATATCCTCAGTTTAGCTTTTTTTACG
460	ATAAGAATGCGGCCGCAGCGATGCTAATCTATGTACACTACGTAAATATC
488	ATCTCTACATACTTGTATATACCGAACATAAGAAGCTCTTAGATTGTACTGAGAGTGCAC
489	TAACTAAAAGTATATATTTGACCAATACCTGACATATCTTCTGTGCGGTATTTCACACCG
535	TGGTTATGCCTTCACCATGA
536	TGAACCATAAACACCATCAGAGA
537	GGTGCCAAGAAGGTTGTCAT
538	AACGGCATCTTCGGTGTAAC
585	AAAGACGCAACGGTCAAGGCTTTCCACGAGACGTTCGAATAGATTGTACTGAGAGTGCAC
586	TATTGTTCTTAATTTACTTAGAGTTATTTAGTTTTTTTAACTGTGCGGTATTTCACACCG
